# The vaccinia virus protein, C16, promotes the ubiquitylation and relocalization of the antiviral E3 ubiquitin-ligase, TRIM25

**DOI:** 10.1128/jvi.00898-25

**Published:** 2025-07-28

**Authors:** Jianing Dong, Shu Yue Luo, Summer Smyth, Grace Melvie, Olivier Julien, Robert J. Ingham

**Affiliations:** 1 Department of Medical Microbiology and Immunology, Li Ka Shing Institute of Virology (LKSIoV), and Striving for Pandemic Preparedness – The Alberta Research Consortium (SPP-ARC), Katz Group Centre for Pharmacy and Health Research, University of Alberta 3158, Edmonton, Alberta, Canada; 2 Department of Biochemistry, LKSIoV, and SPP-ARC, University of Alberta 3158, Edmonton, Alberta, Canada; Northwestern University Feinberg School of Medicine, Chicago, Illinois, USA

**Keywords:** poxvirus, vaccinia virus, ubiquitylation, TRIM25, E3-ubiquitin ligase, diGly

## Abstract

Poxviruses co-opt the ubiquitin (Ub)-proteasome system (UPS) to facilitate virus replication, evade the innate immune response, and block programmed cell death of infected cells. Moreover, the UPS is an integral component of innate immune signaling pathways used by the host to respond to infection. To further elucidate how the UPS is engaged early during poxvirus infection, we quantified viral and cellular peptides with a Ub remnant motif (diGly peptides) from lysates of uninfected and vaccinia virus Copenhagen strain (VACV-Cop)-infected HeLa cells. Of note, we identified several ubiquitylated peptides from the cellular antiviral protein, TRIM25, that were enriched for, or exclusively found, in VACV-Cop-infected cells. TRIM25 is an E3 ligase for Ub and the Ub-like protein, ISG15, and TRIM25 performs several functions including activating the type I interferon response. Higher-molecular weight, ubiquitylated TRIM25 species were evident as early as 1 h post-infection of HeLa cells with VACV-Cop, and they persisted throughout infection. Proteasomal or lysosomal degradation did not appear to be a major consequence of this ubiquitylation; however, TRIM25 ubiquitylation correlated with its relocalization to punctate structures in infected cells. C16, a Bcl-2 family-like protein encoded by identical genes on both arms of the VACV-Cop genome, was both necessary and sufficient to promote TRIM25 ubiquitylation and relocalization. These phenomena were not evident in cells infected with Orthopoxviruses lacking the genes encoding for C16. We postulate that the ubiquitylation and/or relocalization of TRIM25 induced by C16 could represent a novel mechanism for poxviruses to subvert the host antiviral response.

**IMPORTANCE:**

Ubiquitylation is a versatile post-translational modification that is required for poxviruses to replicate their genomes and evade host cell defenses to infection. At the same time, both degradative and non-degradative protein ubiquitylation are critical components of the innate and adaptive immune responses to infection. In this study, we opted for a proteomics approach to examine changes in protein ubiquitylation early after vaccinia virus infection with the goal of identifying novel ways by which ubiquitylation is exploited during infection. We demonstrate that many Orthopoxviruses utilize the Bcl-2 family-like protein C16 to promote the ubiquitylation and relocalization of the cellular E3 ubiquitin/ISG15-ligase, TRIM25, which we hypothesize represents a novel strategy by which these viruses evade the host cell antiviral response. Moreover, our findings hint that Orthopoxviruses may also have C16-independent strategies to interfere with the function of TRIM25.

## INTRODUCTION

Poxviruses are large double-stranded DNA viruses that have broad (e.g., cowpox virus (CPXV)) or narrow (e.g., variola virus (VARV)) host ranges (1, 2). They include important human pathogens such as VARV, the causative agent of smallpox, which killed hundreds of millions of people before its eradication (3–5), and MPox virus (MPXV) responsible for the recent global Mpox outbreak (6). Moreover, some poxviruses (e.g., goat pox, sheep pox, and CPXV) are important livestock pathogens (7, 8), and poxviruses are being developed for use as vaccines (9–13) and oncolytic cancer therapies (14, 15). Thus, there is considerable interest in understanding how poxviruses interact with their hosts during infection. This includes elucidating how poxviruses co-opt and exploit the host cell ubiquitin (Ub)-proteasome system (UPS).

Ubiquitylation is the post-translational modification of proteins with the 76-amino acid protein, Ub. The process starts with the ATP-dependent activation of Ub by an E1 Ub-activating enzyme, followed by the transfer of the Ub to an E2 Ub-conjugating enzyme. The E2 enzyme then facilitates the transfer of the Ub to substrates, directly or indirectly, through binding an E3 Ub-ligase protein, which brings both E2 and the substrate together (16, 17). Proteins can be modified by Ub in a variety of ways. A single Ub molecule can be added to one or more lysine residues in a target protein. Additionally, chains of Ub, formed by Ub molecules being added to lysine residues in Ub itself, can also form on substrates (18). Depending on the lysine residue in Ub used, this generates chains with different topologies and functions. For example, chains generated through the addition of Ub to lysine 48 of Ub (K48-linked polyubiquitin chains) primarily serve as a signal for targeting proteins to the 26S proteasome for degradation (19). In contrast, chains generated through the addition of Ub molecules to lysine 63 (K63-linked polyubiquitin chains) alter protein function and mediate protein-protein interactions (20). Thus, ubiquitylation is an extremely versatile modification that serves numerous functions in the cell. Not surprisingly, poxviruses have developed several strategies to co-opt the host UPS for their benefit.

Poxviruses require the 26S proteasome (21–23) and cellular ubiquitylation components (e.g., E1 Ub-activating enzyme [21, 24] and Cul3 [24]) to productively infect cells. In addition, these viruses encode within their genomes E3 Ub-ligases and substrate adapters for multi-subunit cellular E3 Ub-ligases. These proteins have been implicated in counteracting the innate and adaptive immune responses (25–29), blocking necroptotic cell death (30), and facilitating virus spread (31).

While poxviruses utilize the UPS to successfully establish infection, this system is also important for the host immune response to infection. For example, innate antiviral signaling pathways, such as those involved in the activation of RIG-I (32, 33), and NF-κB (34, 35), utilize degradative and non-degradative ubiquitylation. The UPS is also an important part of the adaptive immune response by processing viral peptides for presentation in major histocompatibility complex (MHC) class I (36).

We were interested in understanding how the UPS is used by the virus and host during the early stages of poxvirus infection. To that end, we identified and quantified changes in diglycine (diGly) peptides, corresponding to ubiquitylation sites, during vaccinia virus Copenhagen strain (VACV-Cop) infection of HeLa cells. In particular, several ubiquitylated peptides from the cellular TRIM25 protein were enriched for, or exclusively identified in, VACV-Cop-infected cells compared to uninfected cells. This observation was of considerable interest given that TRIM25 is an E3 ligase for Ub and the Ub-like protein, ISG15, and one function of this protein is to activate the type I interferon response. We hypothesized that TRIM25 ubiquitylation may represent a novel strategy for VACV-Cop to subvert the host antiviral response. Here, we show that the viral Bcl-2 family-like protein, C16, was both necessary and sufficient to promote TRIM25 ubiquitylation. Proteasomal or lysosomal degradation does not appear to be a major consequence of this ubiquitylation; rather, TRIM25 ubiquitylation correlated with the C16-dependent relocalization of TRIM25 into punctate structures. We postulate that TRIM25 ubiquitylation and/or relocalization could represent viral strategies to counter its antiviral activity.

## MATERIALS AND METHODS

### Antibodies and other reagents

The anti-VACV I3 antibody (Ab) was obtained from Dr. David Evans (University of Alberta). The anti-A34 Ab was a gift from Dr. Bernard Moss (National Institute of Allergy and Infectious Disease), and the anti-B5 Ab was from Dr. Stuart Isaacs (University of Pennsylvania). The anti-β-actin Ab (clone AC-15, #A5441) was purchased from Millipore Sigma. The anti-TRIM25 (#ab167154) and anti-Cul1 (#ab75817) Abs were purchased from Abcam. The anti-conjugated Ub Ab, FK2, was purchased from Enzo Life Sciences (#BML-PW8810), and the P4D1 clone was purchased from Active Motif (#39741). The anti-Myc Ab 9E10 (SC-40) and ISG15 (F-9) were purchased from Santa Cruz Biotechnology Inc. The rabbit monoclonal anti-HJURP Ab (#703460, Invitrogen) was used as an isotype control for anti-TRIM25 immunoprecipitates. The goat anti-rabbit IRDye 680 (#92668071) and goat anti-mouse-IRDye 800 (#92632210) Abs were purchased from LI-COR Biosciences. The secondary antibodies used for immunofluorescence were goat-anti-rabbit Alexa Fluor 647 (#11-605-144, Jackson ImmunoResearch) and goat-anti-mouse Alexa Fluor 488 (#A11001, ThermoFisher).

### Cell lines

HeLa cells were obtained from Dr. Jim Smiley (University of Alberta), and 293T cells were obtained from the American Type Culture Collection (ATCC). BGMK cells (buffalo green monkey kidney cells) were obtained from Dr. David Evans (University of Alberta). These cells were maintained in Dulbecco’s modified Eagle medium (DMEM; #D5796-500ML, Sigma), supplemented with 10% fetal bovine serum (FBS; F1051-500ML, Sigma-Aldrich) and 1% antibiotic-antimycotic solution (#15240062, Gibco). African green monkey kidney epithelial cells (BSC-40) were obtained from the ATCC and grown in minimal essential medium (MEM, #M4655-500ML, Sigma) containing 5% FBS, 1% non-essential amino acids (#11140050, Gibco), 2 mM L-glutamine (#25030081, Gibco), 1 mM sodium pyruvate (#11360070, Gibco), and 1% antibiotic-antimycotic solution. Telomerase reverse transcriptase-immortalized human fetal foreskin fibroblast (HFFF-TERT) cells were a gift from Dr. Victor DeFilippis (Oregon Health & Science University) and maintained in DMEM, supplemented with 10% FBS and 1% antibiotic-antimycotic solution. All cells were incubated at 37°C and 5% CO_2_.

### Viruses

VACV Copenhagen (VACV-Cop) and VACV International Health Department-White (VACV-IHD-W) strains as well as ectromelia virus Moscow (ECTV-Mos) strain were obtained from Dr. Michele Barry (University of Alberta). Deletion viruses derived from VACV-Cop (vP811; vP759; and vP796) originally generated by the Paoletti group (37) were also provided by Dr. Michele Barry. VACV strains Western Reserve (WR), Tian Tan, ACAM2000, as well as cowpox virus (CPXV) strain Brighton Red (BR) were provided by Dr. David Evans (University of Alberta). Lysates of MPXV (Clade IIb)-infected HeLa cells were generously provided by Drs. Yi-Chan Lin and David Evans. The C16L knock-out virus (VACV-Cop ΔC16L/B22R) was generated using CRISPR/Cas9 gene editing, as previously described (38). Briefly, crRNAs recognizing C16L/B22R: (5′- /AltR1/rGrArU rUrGrC rArCrG rArArG rUrUrC rUrUrC rGrGrG rUrUrU rUrArG rArGrC rUrArU rGrCrU /AltR2/ −3′) and (5′- /AltR1/rGrGrA rCrCrC rArUrA rGrArG rArArA rGrCrG rCrArG rUrUrU rUrArG rArGrC rUrArU rGrCrU /AltR2/ −3′) and Cas9 (New England BioLabs) were used to digest VACV-Cop genomic DNA. Then, BGMK cells were infected with shope fibroma virus (SFV) and transfected with the digested genomic DNA and C16L/B22R deletion cDNA fragment (GenScript) to disrupt C16L/B22R. Recombinant viruses were propagated and purified in BSC-40 cells. The deletion of C16L/B22R was first identified via PCR and then confirmed through genome sequencing by Plasmidsaurus. Both approaches indicated that VACV-Cop ΔC16L/B22R does not contain either the C16L or B22R genes.

### DiGly peptide enrichment

HeLa cells (2 × 10^7^) were pretreated with 10 µM MG132 (#BMLPI1020005, Enzo, Life Science) for 1 h. Cells were then inoculated with VACV-Cop viruses at a multiplicity of infection (MOI) of 3 for 1 h and incubated for a further 4 h with 10 µM MG132 in an incubator at 37°C and 5% CO_2_. Cells were lysed in freshly prepared 9 M urea lysis buffer (9 M urea, 20 mM HEPES pH 8.0, 1 mM sodium orthovanadate, 2.5 mM sodium pyrophosphate, and 1 mM β-glycerophosphate), gently probe-sonicated, and the lysates were clarified by spin centrifugation at 13,000 rpm for 10 min at 4°C. Proteins were reduced with 10 mM dithiothreitol (DTT) for 45 min at 37°C, alkylated with 30 mM iodoacetamide for 45 min at room temperature, followed by addition of 20 mM DTT. Proteins were then digested with trypsin in a 1:100 enzyme:substrate ratio overnight at 37°C, and trypsin was precipitated in 2% trifluoroacetic acid (TFA). Peptides were desalted using a SOLA HRP SPE cartridge (#60109-001, Thermo Fisher). Desalted and dried peptides were subjected to automated diGly enrichment using the PTMScan Ub remnant motif kit (#59322, Cell Signaling) on a KingFisher Duo Prime. Briefly, 1 mg of peptides was resuspended in 1 mL HS binding buffer #1, and peptide binding with 20 µL of the Ab-bead slurry was done at room temperature for 2 h. The beads were washed 4 x with 1 mL HS IAP wash buffer and twice with 1 mL H_2_O. Peptides were eluted two times with 50 µL 0.15% TFA at room temperature.

### Mass spectrometry analysis

Peptides were desalted with C18 ziptips (#ZTC18S960, Millipore) and recovered in buffer A (0.1% formic acid) prior to mass spectrometry analysis. The samples were analyzed using a Nanoflow-HPLC (Thermo Scientific EASY-nLC 1200 System) coupled to an Orbitrap Fusion Lumos Tribrid Mass Spectrometer (Thermo Fisher Scientific). Reverse-phase separation of the peptides was done with an Aurora Ultimate analytical column (25 cm x 75 µm ID with 1.7 µm media, IonOpticks). Peptides were eluted with a solvent B gradient (80% ACN, 0.1% FA) for 2 h. The gradient was run at 400 nL/min with the analytical column temperature set at 45°C. The data were analyzed using Proteome Discoverer (v2.4.1.15) against the concatenated database of the human proteome (UP000005640) and VACV-Cop proteome (UP000008269), with the relaxed false discovery rate set at 5% and restricted at 1%. Search parameters included a maximum of two missed trypsin cleavages, a precursor mass tolerance of 15 ppm, a fragment mass tolerance of 0.8 Da, with the constant modification carbamidomethylation (C), and variable modifications of acetyl (protein N-term), deamidated (N/Q), oxidation (M), and GlyGly (uncleaved K). The maximum number of variable modifications was set to 4. Statistical analysis was performed by Proteome Discoverer using background-based Student *t*-test, and protein abundance ratios were calculated using pairwise peptide ratios.

### Metascape analysis

diGly peptides enriched in either uninfected or infected cells in at least two independent experiments were analyzed by Metascape (v3.5.20230501) using express analysis and the human species setting. Categories with a significant adjusted *P* value (-log(q) >1.5 or q < 0.05) are shown.

### Examination of cellular protein levels associated with enriched diGly peptides in VACV-infected cells

The data of Soday et al. was analyzed to examine levels of host proteins with enriched diGly peptides during VACV-WR infection (39). The MG132 rescue ratio ($\frac{\text{Protein abundance(VACV infected; +MG132)}/\text{Protein abundance(VACV infected; -MG132)}}{\text{Protein abundance (uninfected +MG132)}/\text{Protein abundance (uninfected; -MG132)}}$) reported in this study was included.

### Transfection and nucleofection of cDNAs

HeLa cells were either transfected with the indicated cDNAs using Lipofectamine 2000 (Invitrogen) or nucleofected using a Lonza Amaxa SE Cell line 4D-Nucleofector X kit (#V4XC-1032) and an Amaxa Nucleofector. The human codon-optimized Myc-C16 cDNA was ordered from GenScript based on the previously described shorter C16 isoform (40). The JunB N-terminal fragment has been previously described (41). Cells were analyzed 16 h after nucleofection or 48 h after transfection.

### Preparation of cell lysates

HeLa cells were either left uninfected or infected with the indicated viruses at an MOI of 10. Cells were then washed with 1 mL phosphate-buffered saline (PBS; #806552-500ML, Millipore Sigma) and lysed in 1% NP-40 lysis buffer (1% NP-40, 50 mM Tris-HCl pH7.4, 150 mM NaCl, 2 mM EDTA, 10% glycerol) containing protease inhibitor cocktail (1 tablet in 10 mL of lysis buffer) (#11836170001, Roche), 1 mM phenylmethylsulfonyl fluoride (PMSF; PMS123.5, Bioshop), 5 mM N-ethylmaleimide (NEM; E3876-5G, Millipore Sigma), and 1 mM sodium orthovanadate (Na_3_VO_4_; S6508-10G, Millipore Sigma) for 10 min on ice. The detergent-insoluble material was removed by centrifugation at 20,000 × *g* for 10 min at 4°C. Lysates were stored at −20°C until used.

### Proteasomal and lysosomal inhibition experiments

HeLa cells were pretreated with 10 µM MG132 (proteasome inhibitor) or 20 mM NH_4_Cl (lysosome inhibitor) in complete media for 1 h at 37°C and 5% CO_2_. Cells were then infected with VACV-Cop at an MOI of 10 for 1 h in serum-free media without the drug. After which, infected cells were incubated at 37°C and 5% CO_2_ in the presence of MG132 or NH_4_Cl for the remainder of infection before lysis.

### UV inactivation of virus

VACV-Cop was diluted in 1 mL DMEM (~2 × 10^7^ pfu/mL) and placed in a 6-well plate before being irradiated with 0.03 J/cm^2^ UV energy using a CL-1000 Ultraviolet Crosslinker (UVP). HeLa cells were then inoculated with VACV-Cop or UV-inactivated virus at an MOI of 10 for the indicated time points at 37°C, after which the virus was removed from the culture and replaced with fresh media. HeLa cells were harvested 4 h post-infection (hpi), and lysates were analyzed by Western blotting using the indicated Abs. In parallel, the titer of VACV-Cop and the UV-inactivated virus was determined in BSC-40 cells, as previously described (42).

### Immunoprecipitation (IP) experiments

Protein lysates were incubated with 5 µg anti-TRIM25 or isotype control (anti-HJURP) Ab at 4°C overnight before being incubated with 10 µL packed Protein A-Sepharose beads (Millipore Sigma) for 2 h at room temperature. Beads were washed with 1 mL of 1% NP-40 lysis buffer twice and boiled in 30 µL of SDS-PAGE sample buffer to elute bound proteins. For the anti-conjugated Ub IPs, 2 µg FK2 Ab was incubated with 10 µL packed Protein A/G-Agarose beads (#20423, Thermo Fisher Scientific) for 2 h at room temperature. Beads were washed once with 1 mL of 1% NP-40 lysis buffer and then incubated with protein lysates at 4°C overnight. Beads were then washed three times with 1 mL of 1% NP-40 lysis buffer, and bound proteins were eluted with 30 µL of SDS-PAGE sample buffer. For the anti-Myc IPs, 2 µg anti-Myc Ab (9E10) was incubated with 10 µL packed Protein A/G-Agarose beads for 2 h at room temperature. Beads were washed once with 1 mL of 1% NP-40 lysis buffer and then incubated with protein lysates at 4°C overnight. Beads were then washed six times with 1 mL of 1% NP-40 lysis buffer, and bound proteins were eluted with 30 µL of SDS-PAGE sample buffer.

### Deubiquitylation experiments

Deubiquitylation assays were performed as previously described (43). Samples from anti-conjugated Ub IP were washed and incubated in deubiquitylation buffer (50 mM NaCl, 50 mM Tris, pH 7.4, and 50 mM DTT) with 10 µM USP2 protein (R&D systems, #E-504) at 30°C for 2 h. To stop the reaction, samples were boiled with sample buffer for 10 minutes. Western blot analysis using the indicated antibodies was then performed.

### Ubiquitylation and neddylation inhibitor treatments

HeLa cells were pretreated with 1 µM TAK-243 (#S8341, SelleckChem) for 4 h or the indicated concentrations of MLN4924 (#12.05477.0001, EMD Millipore Corp) for 20 h. The medium was removed, and cells were infected with VACV-Cop at an MOI of 10 for 1 h. The virus inoculum was aspirated and replaced with fresh medium containing the indicated drugs for 3 h. Cells were then lysed, and lysates were separated by SDS-PAGE before being subjected to Western blotting.

### Western blotting experiments

Protein lysates or IPs were separated by SDS-PAGE (Hoeffer) before being transferred to nitrocellulose membranes (#1620115, Bio-Rad) using a semi-dry transfer apparatus (Bio-Rad). Membranes were then blocked with 5% milk in PBS before being incubated with the indicated primary Abs diluted in PBS. Membranes were then incubated with secondary Abs, goat-anti-rabbit 680 or goat-anti-mouse 800, for 45 min at room temperature in the dark, washed 3 × 5 min with PBST, and imaged using an Odyssey scanner (LI-COR Biosciences).

The levels of immunoreactive bands in Western blotting experiments were determined using Image Studio 5.5.4. TRIM25 protein levels were normalized to the level of β-actin in the lane. All quantification, except that in Fig. 4, was made relative to uninfected cells, which were arbitrarily set at 1. In Fig. 4, quantification was made relative to infected cells not treated with MG132.

### Immunofluorescence microscopy

HeLa cells were seeded on flamed glass coverslips placed in a 24-well plate and infected with the indicated viruses at an MOI of 3 diluted in serum-free DMEM. After 1 h in the 37°C, 5% CO_2_ incubator, the inoculum was replaced with complete media. At the indicated time point, the glass coverslip was washed with PBS once, fixed with 4% PFA for 20 min at room temperature, quenched with 250 mM glycine for 5 min, washed three times with PBST, and permeabilized with 0.1% Triton X-100 in PBS for 2 min. Samples were then blocked with 1:1 Odyssey blocking buffer: PBS overnight at 4°C. The coverslips were then incubated with primary antibodies overnight at 4°C. The next day, the samples were incubated with secondary antibodies for 1 h, before DAPI (#28718-90-3, Sigma-Aldrich) was added at 5 µg/mL for 15 min at room temperature. Samples were washed three times with PBST, once with PBS, and then mounted in mowiol mounting media at 56°C onto microscope slides.

Slides were visualized on a WaveFX spinning disk confocal microscope (Quorum Technologies) fitted with a 100X/1.4 oil lens, using the Cy5, FITC, and DAPI lasers. Z-stack images were taken at a spacing of 0.2 µM for every cell. Imaging was done using a Hamamatsu EMCCD (C9100-13) or Hamamatsu Orca-Fusion BT Digital sCMOS cameras, and acquisition was performed using Volocity (PerkinElmer). Presented images represent merged Z stacks.

### Puncta and colocalization quantification

Punctae were quantified using the ImageJ Fiji FindFoci plugin, as described by 44. Quantification represents foci/punctae above a threshold brightness. The percentage overlap of channels was analyzed using the ImageJ Fiji JACoP plugin, as described by 45. Thresholding was done for each channel until foci could be illuminated from the background, and colocalization was measured as the percentage signal overlap.

### Generation of ISG15 and TRIM25 knock-out HeLa cells

TRIM25 knock-out cells were generated using CRISPR, as previously described (46). The crRNAs targeting TRIM25 (5′- /AltR1/rCrArC rCrArA rGrCrA rCrGrU rCrUrU rCrArC rGrGrG rUrUrU rUrArG rArGrC rUrArU rGrCrU /AltR2/ −3′) and ISG15 crRNA#1 (5′- /AltR1/rCrGrU rCrUrG rGrCrU rGrUrC rCrArC rCrCrG rArGrG rUrUrU rUrArG rArGrC rUrArU rGrCrU /AltR2/ −3′) ISG15 crRNA#2 (5′- /AltR1/rGrGrA rCrCrU rGrArC rGrGrU rGrArA rGrArU rGrCrG rUrUrU rUrArG rArGrC rUrArU rGrCrU /AltR2/ −3′) were designed using the IDT design tool. The Alt-R CRISPR-Cas9 negative control crRNA #1 was used as a control cRNA (#1072544, IDT). Alt-R CRISPR-Cas9 trans-activating crRNA (tracrRNA) (#1072532, IDT) was mixed with crRNA and then incubated with Alt-R *S.p*. Cas9 nuclease V3 (#1081059, IDT). The RNA/Cas9 complex was nucleofected into HeLa cells using a Lonza Amaxa SE Cell line 4D-Nucleofector X kit (#V4XC-1032) and an Amaxa Nucleofector Device. Transfected cells were incubated for 24 h, and individual clones were selected by limiting dilution, and knock-outs were confirmed by Western blotting.

### Multi-step growth curve experiments

HeLa or HFFF-TERT cells were inoculated with the indicated viruses in serum-free media at an MOI of 0.03 for 1 h. Then, the inoculum was aspirated and replaced with complete media. Infected cells were harvested with a cell scraper at the indicated time points. Virus particles were released by 3 x freeze-thaw, and viral titers were determined on BSC-40 cells, as previously described (42).

### Statistics

When comparing two samples, one-tailed, paired Student *t*-tests were performed. One- and two-way analysis of variance (ANOVA) was performed when comparing more than two samples. A *P* value ≤ 0.05 was considered statistically significant.

## RESULTS

### Identification of diGly peptides from uninfected and VACV-Cop-infected HeLa cells

**Fig 1:**
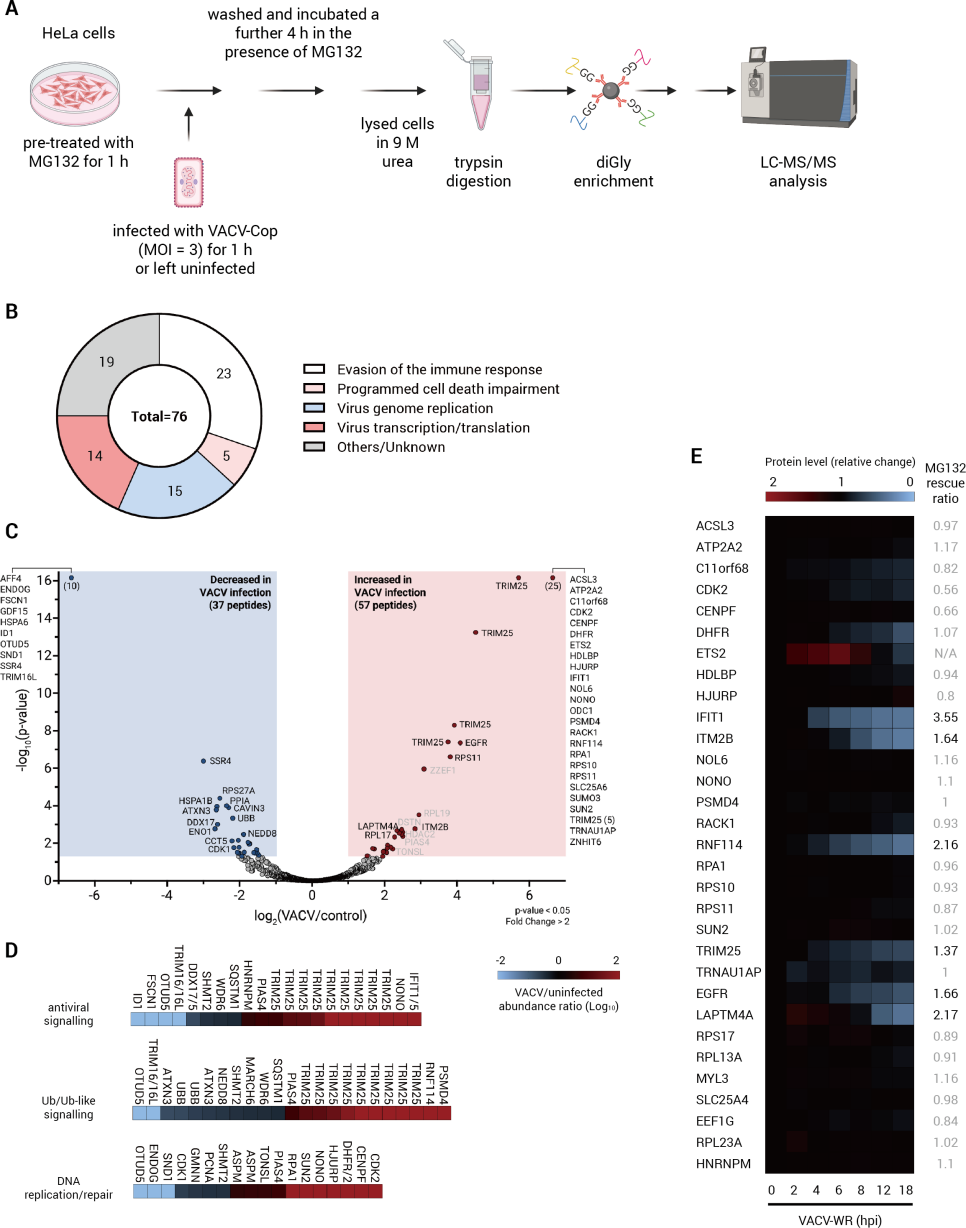
Identification of diGly peptides in VACV-Cop-infected HeLa cells. (A) Outline of the experimental workflow used for the purification and identification of diGly peptides. (B) Summary of the number of viral proteins with the indicated functions for which diGly peptides were identified in at least two independent experiments. (C) Volcano plot of diGly peptides derived from host proteins found in at least two independent experiments. Peptides in blue were enriched ≥2 fold (*P* value ≤ 0.05) in uninfected cells, whereas peptides in pink were enriched ≥2 fold (*P* value ≤ 0.05) in VACV-Cop-infected cells. Peptides in gray were considered of lower confidence given their prominent enrichment in only one of the replicates. (D), Heatmap of identified diGly peptides from proteins associated with antiviral signaling, Ub or Ub-like signaling, or DNA replication/repair, was determined from the literature. The abundance ratio (VACV-infected/-uninfected; Log_10_ scale) is indicated. Peptides with an abundance ratio of −2 were exclusively found in uninfected cells, whereas those with an abundance ratio of 2 were found exclusively in infected cells. (E), Proteins with diGly peptides enriched in VACV-Cop-infected HeLa cells were searched in the data set generated by Soday et al. (39), which examined changes in protein levels over the course of VACV-WR infection of HFFF-TERT cells. The heatmap shows the changes in protein levels during the infection relative to the uninfected sample, which was arbitrarily set at 1. MG132 rescue ratio, as determined by (39), is indicated. Those with a rescue ratio greater ≥1.25 are indicated in black.

To investigate changes in protein ubiquitylation early during poxvirus infection, HeLa cells were pretreated with the proteasome inhibitor, MG132, for 1 h and either left uninfected or infected with VACV-Cop at an MOI of 3 for 1 h, followed by an additional 4 h incubation in the presence of MG132 (Fig. 1A). Lysates were then subjected to trypsin digestion, and diGly peptides were enriched for before being analyzed by liquid chromatography, followed by tandem mass spectrometry (LC-MS/MS). diGly enrichment utilizes an Ab that recognizes the diGly remnant present on ubiquitylated lysine residues after trypsin digestion (47, 48). Three independent experiments identified 1,753 (1,494 cellular and 259 viral) distinct diGly peptides (Fig. S1A and Table S1). The majority of peptides (≥78%) were identified in both uninfected and infected cells (Fig. S1B), albeit their abundance was not necessarily the same. Moreover, there was considerable overlap in diGly peptides identified between the independent experiments (Fig. S1C).

We focused our analysis on the 1,720 diGly peptides found in at least two independent experiments. Quantifying average peptide abundance over the three independent experiments revealed 304 peptides enriched for (≥ 2 fold change and *P* ≤ 0.05) in VACV-Cop-infected cells (Table S2). The majority of these diGly peptides (247; 81%) were from viral proteins (76 in total) and included proteins associated with virus genome replication, inhibition of programmed cell death, manipulation of host immune pathways, and viral transcription/translation (Fig. 1B). Fifty-seven peptides derived from cellular proteins were enriched in infected cells, whereas 37 cellular diGly peptides were enriched in uninfected cells (≥ 2 fold change and *P* ≤ 0.05) (Fig. 1C; Table S3).

Metascape analysis was performed to determine whether any cellular pathways or processes were overrepresented from proteins associated with diGly peptides predominantly found in uninfected or infected cells. Translation and the cell cycle were categories associated with proteins with peptides enriched in infected cells (Fig. S2A), whereas processes linked to proteins with peptides enriched in uninfected cells included deubiquitylation, viral infection pathways, DNA replication, and necroptosis (Fig. S2B). Since these analyses may not include all known functions attributed to these proteins, we examined the literature to identify additional activities regulated by these proteins. We found that several of the peptides were from proteins associated with antiviral signaling, Ub/Ub-like signaling, and DNA replication/repair (Fig. 1D). In addition, several peptides were from proteins previously implicated in poxvirus infection including IFITs (28), WDR6 (49), RACK1 (50), DDX5 (51), and EGFR (52).

The identification of a remnant diGly lysine residue on a peptide is indicative of the site being ubiquitylated. However, it does not provide any information about the consequence of this ubiquitylation. Therefore, we compared our data to published work that analyzed changes in protein levels in telomerase reverse transcriptase (TERT)-immortalized human fetal foreskin fibroblast (HFFF-TERT) cells infected with VACV Western Reserve strain (VACV-WR) (39). Despite this study using a different cell line and VACV strain, several of the proteins with diGly peptides enriched in VACV-Cop-infected HeLa cells were decreased over the course of infection (Fig. 1E). Furthermore, this study showed that protein levels for most of the downregulated proteins could be partially rescued by treatment with MG132, suggesting they are likely degraded in a proteasome-dependent manner (Fig. 1E), which has been independently confirmed for the IFIT proteins (28). Intriguingly, the levels of many proteins with diGly peptides enriched in VACV-Cop-infected HeLa cells were not decreased in HFFF-TERT cells infected with VACV-WR, suggesting ubiquitylation of some of these proteins could serve a non-degradative function (Fig. 1E).

### VACV-Cop infection resulted in the formation of higher-molecular weight (HMW) TRIM25 species and a decrease in TRIM25 immunoreactive protein levels

**Fig 2:**
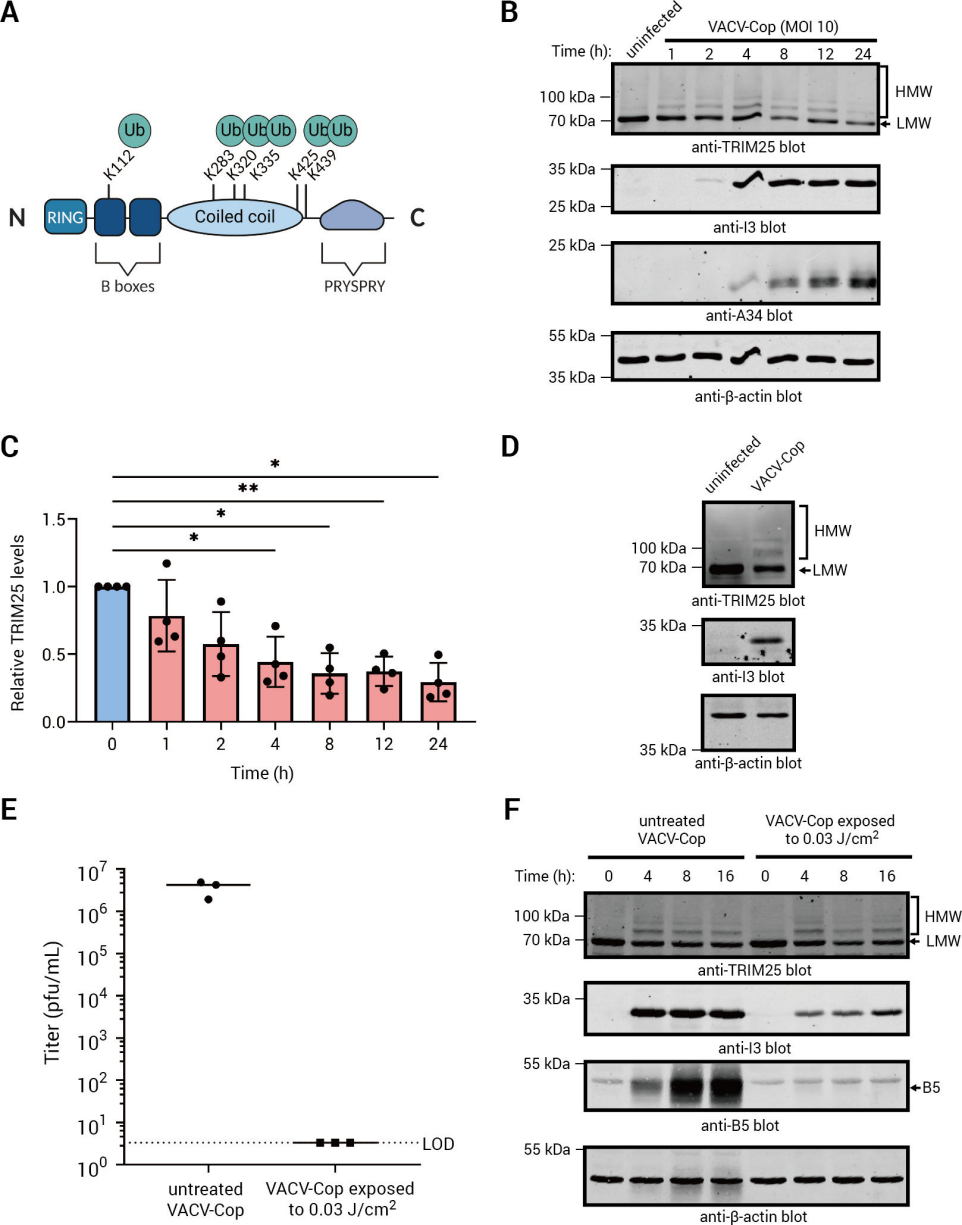
VACV-Cop infection resulted in the formation of TRIM25 HMW species and decreased TRIM25 detection. (A) Cartoon showing the location of identified TRIM25 diGly peptides. The relative position of TRIM25 domains was obtained from Koliopoulos et al. (64). (B) Lysates of uninfected HeLa cells or HeLa cells infected (MOI of 10) for the indicated time points with VACV-Cop were immunoblotted with Abs against the indicated proteins. I3 and A34 are viral proteins first expressed either at the early or late stage of virus infection, respectively. Western blots of these proteins were included to show the course of infection. The anti-β-actin blots were included to show protein loading. (C) Quantitative analysis of TRIM25 immunoreactive bands in VACV-Cop-infected HeLa cells (mean and standard deviation) relative to uninfected cells from four independent experiments. A one-way ANOVA was used to calculate statistical significance between uninfected cells (0 h time point) and the indicated time points post-infection. *; *P* ≤ 0.05, **; *P* ≤ 0.01. (D) 293T cells were either left uninfected or infected with the VACV-Cop for 4 h (MOI of 10). Lysates were immunoblotted with Abs against the indicated proteins. (E) Titers of VACV-Cop in BSC-40 cells exposed to the indicated UV light energy compared to the untreated virus. The dotted line indicates the limit of detection (LOD). Results presented represent the mean and standard deviation from three independent experiments. (F) Western blot analyzing TRIM25 in HeLa cells infected with VACV-Cop or UV-inactivated VACV-Cop for the indicated time points. The anti-B5 blot was included to show that expression of the B5 late gene was absent in cells treated with the UV-inactivated virus. For all blots, molecular mass markers are indicated to the left of blots.

The most striking observation from the diGly enrichment data was the identification of nine diGly peptides from TRIM25 (Fig. 1C), that could be definitively assigned to six lysine residues (Fig. 2A), that were enriched for or exclusively found in VACV-Cop-infected HeLa cells. Moreover, TRIM25 levels were found to decrease ~40% in a proteasome-dependent manner over the course of infection of HFFF-TERT cells with VACV-WR (Fig. 1E) (39). TRIM25 is a really interesting new gene (RING) domain-containing E3 Ub-ligase (53) that also functions as an E3 ligase for the Ub-related molecule, ISG15 (54). TRIM25 performs many functions (55), but is perhaps most well-known for its role in antiviral signaling. For example, TRIM25 introduces K63-linked Ub chains onto RIG-I (56, 57). RIG-I is a pattern recognition receptor that recognizes viral RNA and initiates signals that lead to the production of type I interferons (reviewed in [58]). Rather than promoting RIG-I degradation, ubiquitylation by TRIM25 promotes RIG-I activation (56). It is therefore not surprising that many viruses have acquired mechanisms to interfere with the ability of TRIM25 to ubiquitylate RIG-I ([59–61] as examples). In a similar fashion, TRIM25 ubiquitylates zinc-finger antiviral protein (ZAP), which enhances ZAP-mediated inhibition of viral transcription and mRNA translation (62, 63).

To further examine whether VACV-Cop infection of HeLa cells had any consequence on the TRIM25 protein, we performed Western blotting experiments. We chose to increase the MOI in these experiments to 10 to ensure all cells were infected. These experiments revealed the appearance of HMW, potentially ubiquitylated, TRIM25 species and a decrease in the lower-molecular weight (LMW) TRIM25 band over the course of infection (Fig. 2B). HMW TRIM25 proteins were evident as early as 1 hpi, persisted throughout infection, but were reduced at 24 hpi (Fig. 2B). Quantification of total anti-TRIM25 immunoreactive bands in uninfected and infected cells showed a decrease in anti-TRIM25 levels over the course of infection (Fig. 2C). Similar results were observed in VACV-Cop-infected 293T cells (Fig. 2D). Moreover, treatment of HeLa cells with UV-inactivated VACV-Cop, which was unable to replicate (Fig. 2E), was still able to induce TRIM25 HMW species (Fig. 2F). This demonstrates that replication-competent virus is not required for this modification of TRIM25.

### TRIM25 HMW species are ubiquitylated forms of the protein

**Fig 3:**
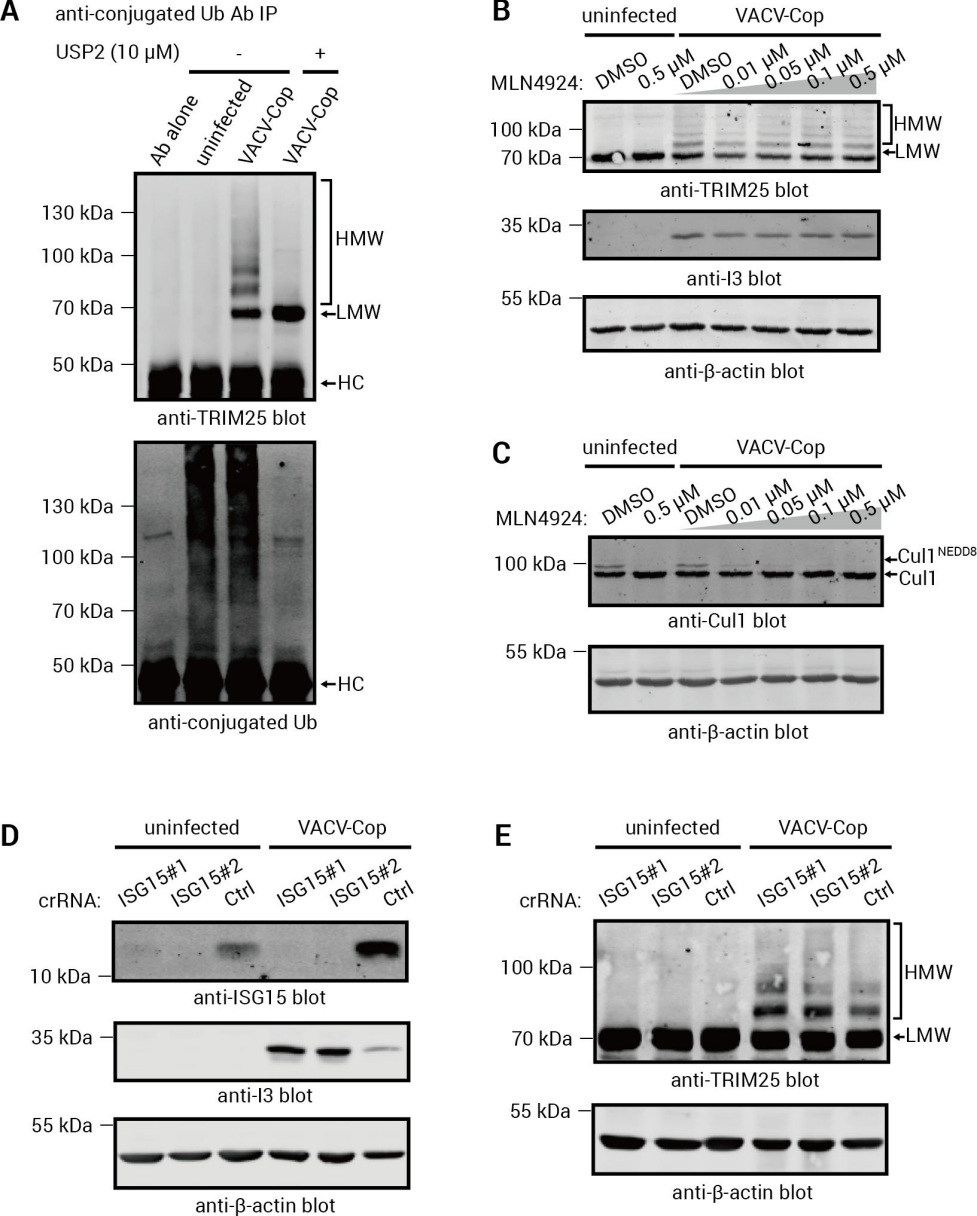
TRIM25 was ubiquitylated in VACV-Cop-infected cells. (A) Cell lysates from uninfected HeLa cells or HeLa cells infected for 4 h with VACV-Cop (MOI of 10) were used to perform IPs with an anti-conjugated Ub Ab, and IPs were then treated with (+) or without (−) the USP2 deubiquitinase before being immunoblotted for TRIM25 (upper panel) or conjugated Ub. HC = heavy chain of immunoprecipitating Ab. (B and C) Lysates were prepared from HeLa cells treated with the indicated concentrations of MLN4924 and either left uninfected or infected with VACV-Cop for 4 h (MOI of 10). Lysates were then immunoblotted with Abs against the indicated proteins. The anti-Cul1 Ab recognizes both unmodified Cul1 and NEDD8-modified (Cul1^NEDD8^). (D and E) Lysates from uninfected or VACV-Cop-infected (4 h; MOI of 10) HeLa cells or HeLa cells lacking ISG15 were immunoblotted with Abs against the indicated proteins. The two ISG15 knock-out clones (ISG #1 and #2) were generated using different crRNAs. Molecular mass markers are indicated to the left of blots.

To directly examine whether TRIM25 was ubiquitylated in VACV-Cop-infected cells, we immunoprecipitated (IPed) TRIM25 from lysates of uninfected or VACV-Cop-infected HeLa cells and immunoblotted with an anti-Ub Ab. Although there were faint bands that could represent ubiquitylated TRIM25 species, the results of these experiments were inconclusive, likely due to an inability to efficiently IP TRIM25, especially the HMW forms (Fig. S3). Therefore, we used an Ab that recognizes conjugated Ub to IP ubiquitylated proteins from cells and examined whether this enriched for HMW TRIM25 species. TRIM25 HMW species were observed in the anti-conjugated Ub IPs, consistent with these being ubiquitylated forms of the protein (Fig. 3A; upper panel). Moreover, treating the IPs with USP2, a broad specificity deubiquitylase (43, 65, 66), resulted in the disappearance of the TRIM25 HMW species and an increase in the LMW TRIM25 band. This demonstrated that these TRIM25 HMW species are ubiquitylated forms of the protein.

While the vast majority of diGly sites are derived from ubiquitylation (67), proteins modified on lysine residues with the Ub-like proteins, NEDD8 and ISG15, can also generate diGly peptides after trypsin digestion (47, 67). Therefore, we examined whether the formation of HMW TRIM25 species was affected by treatment with the neddylation E1 protein inhibitor, MLN4924. No decrease in HMW TRIM25 species was observed in cells treated with MLN4924 (Fig. 3B). However, neddylation of a known NEDD8 modified protein, Cul1 (68), was affected by MLN4924 treatment (Fig. 3C). We also used clustered regularly interspaced short palindromic repeats (CRISPR)/Cas9-mediated gene editing to delete ISG15 from the genome of HeLa cells (Fig. 3D) and found that this had no observable impact on the formation of TRIM25 HMW species in infected cells (Fig. 3E). Taken together, these experiments demonstrate that TRIM25 HMW species are predominantly ubiquitylated forms of the protein.

### Neither proteasomal- nor lysosomal-mediated degradation appears to be a major consequence of TRIM25 ubiquitylation

**Fig 4:**
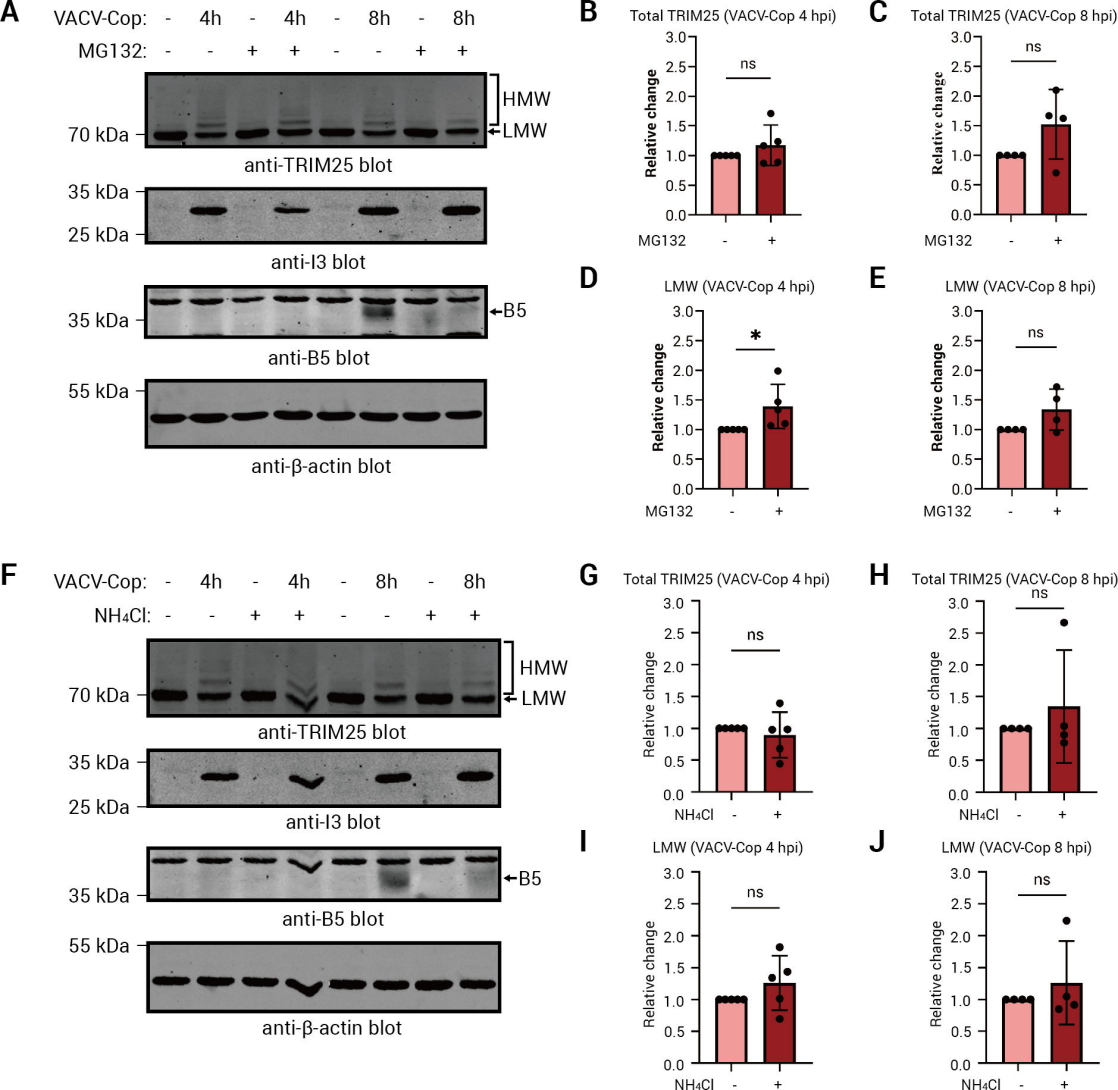
Neither proteasomal- nor lysosomal-mediated degradation appears to be a significant cause for the apparent reduction in TRIM25 levels. (A) HeLa cells were treated with or without 10 µM MG132 and either left uninfected or infected with VACV-Cop for the indicated time points. Lysates were immunoblotted for Abs against the indicated proteins. Quantitative analysis of total anti-TRIM25 immunoreactive bands 4 (**B**) or 8 (**C**) hpi in cells infected in the presence (+) or absence (−) of MG132 is shown. Quantification is from 5 (**B**) or 4 (**C**) independent experiments and expressed relative to the samples not treated with MG132. The abundance of the LMW TRIM25 band 4 (**D**) or 8 (**E**) hpi is shown. Quantification represents the mean and standard deviation from 5 (**D**) or 4 (**E**) independent experiments and is expressed relative to the samples not treated with MG132. F, HeLa cells were treated with or without 20 mM NH_4_Cl and either left uninfected or infected with VACV-Cop for the indicated time points. Quantitative analysis of total anti-TRIM25 immunoreactive bands 4 (**G**) or 8 (**H**) hpi in cells infected in the presence (+) or absence (−) of NH_4_Cl is shown. Quantification is from 5 (**G**) or 4 (**H**) independent experiments and expressed relative to the samples not treated with NH_4_Cl. The abundances of the LMW TRIM25 band 4 (**I**) or 8 (**J**) hpi are shown. Quantification represents the mean and standard deviation from 5 (**I**) or 4 (**J**) independent experiments and expressed relative to the samples not treated with NH_4_Cl. A paired, one-tailed Student *t*-test was used to calculate statistical significance between groups. ns; not significant, *; *P* ≤ 0.05. Molecular mass markers are indicated to the left of blots.

A proteomic study suggested that TRIM25 was degraded in a proteasome-dependent manner in HFFF-TERT cells infected with VACV-WR (Fig. 1E) (39). To test if this was true in VACV-Cop-infected HeLa cells, we treated cells before and after infection with the proteasome inhibitor, MG132, and TRIM25 levels were examined at 4 and 8 hpi. Total TRIM25 levels were modestly increased in cells treated with MG132 (Fig. 4A), more so at 8 hpi, but these differences were not statistically significant (Fig. 4B and C). Similar observations were made for the LMW TRIM25 species (Fig. 4A and D, and E). MG132 treatment blocked the expression of the B5 viral late protein (Fig. 4A), which is consistent with studies showing that late gene expression requires proteasomal activity (21, 22, 24). Cytosolic ubiquitylated proteins can also be targeted to lysosomes for degradation (69). Therefore, we examined whether lysosomal inhibition impacted TRIM25 levels in VACV-Cop-infected HeLa cells. Similar to what we observed in MG132-treated cells, lysosome inhibition with NH_4_Cl (70, 71) did not markedly rescue TRIM25 levels in VACV-Cop-infected HeLa cells (Fig. 4F through J). Thus, neither proteasomal- nor lysosomal-mediated degradation appears to be a significant cause for the apparent reduction in TRIM25 levels.

### TRIM25 relocalized to punctate structures in VACV-Cop-infected HeLa cells

**Fig 5:**
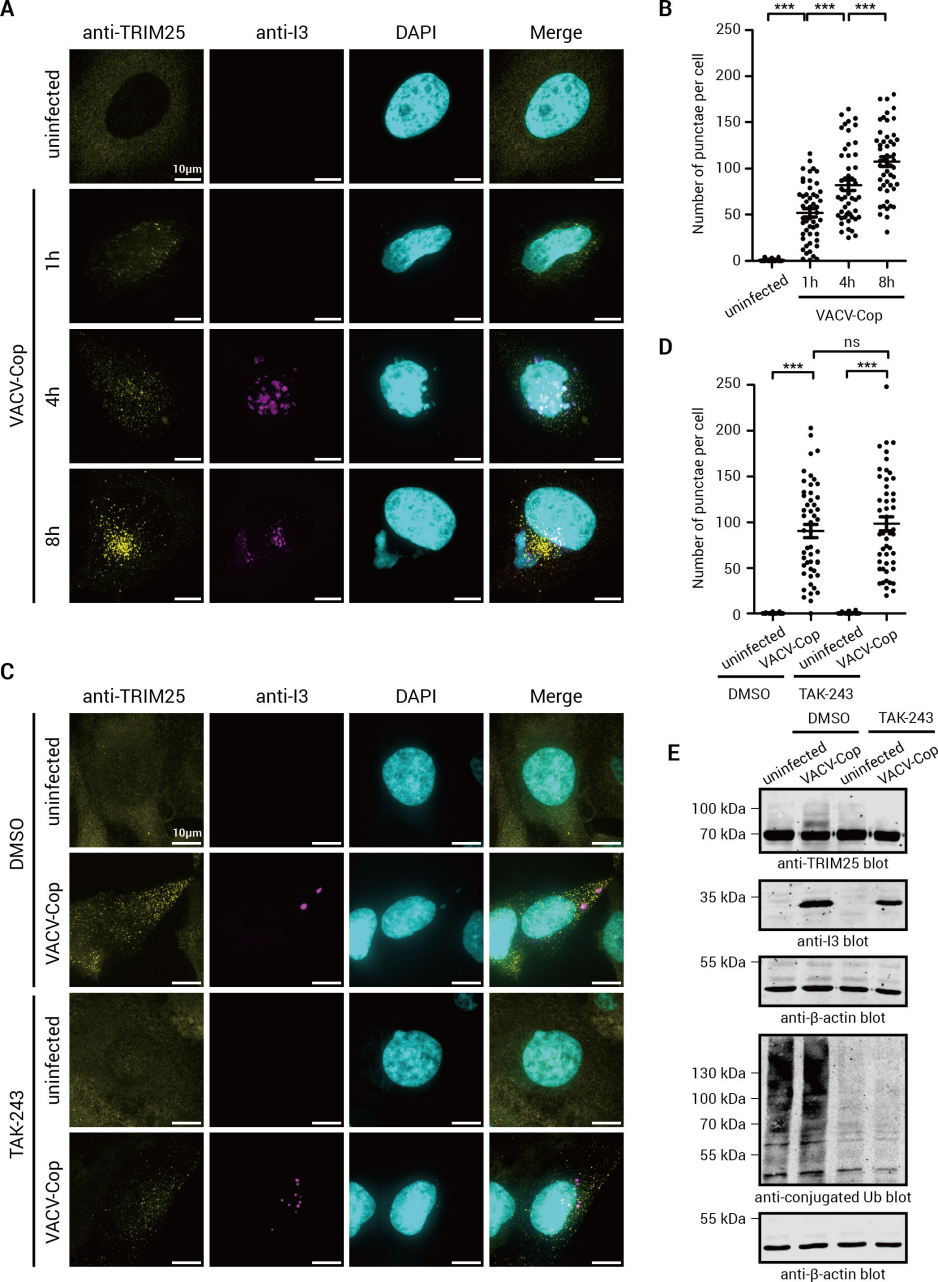
TRIM25 relocalizes to punctate structures in VACV-Cop-infected HeLa cells in a ubiquitylation-independent manner. (A) HeLa cells were either left uninfected or infected with VACV-Cop, at an MOI of 3 for the indicated time points on glass coverslips. Cells were then fixed, permeabilized, blocked, and stained with antibodies against the indicated proteins. DAPI was used to stain DNA. (B) Quantification of the number of TRIM25 punctae/cell in VACV-Cop-uninfected and VACV-Cop-infected cells in (A). The results presented are from two biological replicates with at least 25 randomly chosen cells per treatment from each replicate. Outliers were excluded by IQR score. (C) HeLa cells treated with or without 1 µM TAK-243 were either left uninfected or infected with VACV-Cop (MOI of 3) for the indicated time points. The cells were then stained with DAPI and antibodies against the indicated proteins. (D) Quantification of the number of TRIM25 punctae/cell in VACV-Cop-uninfected and VACV-Cop-infected cells in (C). The results presented are from two biological replicates with at least 25 randomly chosen cells per treatment from each replicate. Outliers were excluded by IQR score. (E) Western blots demonstrating TAK-243 treatment blocked TRIM25 ubiquitylation (upper panel) and total cellular conjugated ubiquitylation (lower panel). Note: The anti-conjugated Ub blot is from the same samples as above run on a different gel. Molecular mass markers are indicated to the left of blots. One-way ANOVA was used to calculate the statistical significance between samples. ns; not significant, ***; *P* ≤ 0.001. The scale bar on images = 10 µm.

Since neither proteasomal- nor lysosomal-mediated degradation appeared to be the major consequence of TRIM25 ubiquitylation (Fig. 4), we investigated whether ubiquitylation may serve another function. Ubiquitylation can mediate protein-protein interactions (72, 73) and regulate the cellular localization of the modified protein (74, 75). Therefore, we examined whether TRIM25 localization was impacted by VACV-Cop infection. In uninfected HeLa cells, TRIM25 staining was diffuse throughout the cytoplasm (Fig. 5A). In contrast, TRIM25 in VACV-Cop-infected cells was found in punctate structures as early as 1 hpi, with the number of these structures increasing over the course of infection (Fig. 5A and B). To examine whether TRIM25 ubiquitylation, and ubiquitylation more generally, was required for TRIM25 relocalization, we treated HeLa cells with the E1 Ub-activating enzyme inhibitor, TAK-243 (76), before infection with VACV-Cop. TAK-243 treatment did not impair TRIM25 relocalization in VACV-Cop-infected cells (Fig. 5C and D), despite blocking the formation of TRIM25 ubiquitylated species and significantly impairing total cellular ubiquitylation (Fig. 5E). Thus, TRIM25 is ubiquitylated and relocalizes to punctate structures in VACV-Cop-infected HeLa cells, but ubiquitylation is not required for its relocalization.

### TRIM25 HMW species and relocalization were observed in HeLa cells infected with some, but not all, Orthopoxviruses

**Fig 6:**
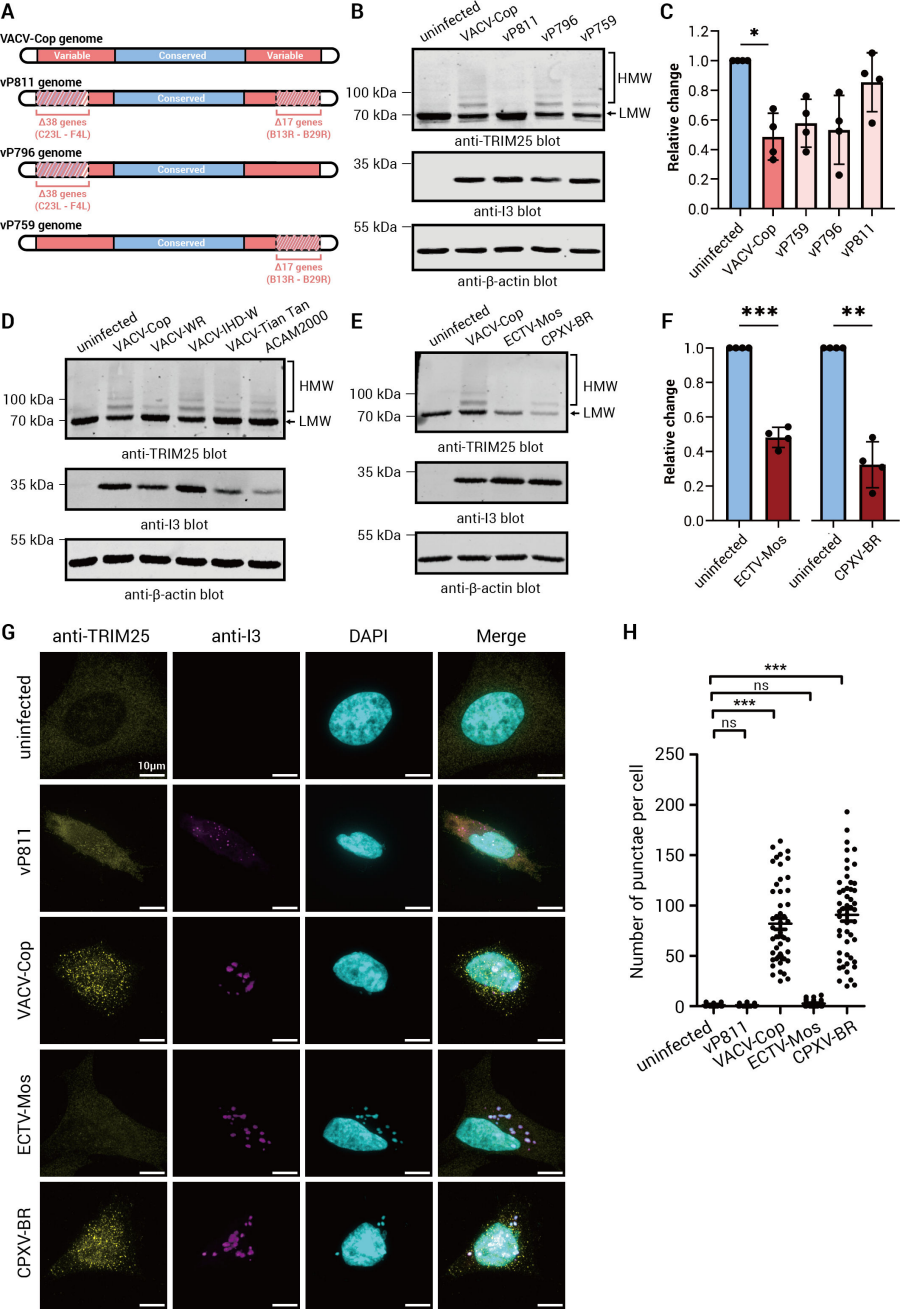
TRIM25 HMW, ubiquitylated species were observed in HeLa cells infected with some, but not all, Orthopoxviruses, and the formation of these species correlated with TRIM25 punctae formation. (A) Cartoons of the genomes of VACV-Cop and the VACV-Cop deletion viruses, vP811, vP796, and vP759. (B) Lysates of HeLa cells either left uninfected or infected (MOI of 10) for 4 h with the indicated viruses were immunoblotted with Abs against the indicated proteins. (C) Quantification analysis of total anti-TRIM25 immunoreactive bands in cells either left uninfected or infected with indicated viruses in (B). Quantification is from four independent experiments and expressed relative to the samples left not infected with viruses. HeLa cells were either left uninfected or infected with the indicated VACV strains (D) or Orthopoxviruses (E) for 4 h (MOI of 10). Lysates were immunoblotted with Abs against the indicated proteins. Results are representative of 3 (D) and 4 (E) independent experiments. (F) Quantification analysis of total anti-TRIM25 immunoreactive bands in cells either left uninfected or infected with indicated viruses in (E). Quantification is from four independent experiments and expressed relative to the samples uninfected with viruses. (G) HeLa cells were either left uninfected or infected with the indicated viruses at an MOI of 3 for 4 h. Cells were then fixed, permeabilized, blocked, and stained with the antibodies against the indicated proteins. DAPI was used to stain DNA. (H) Quantification of the number of TRIM25 punctae/cell in uninfected and cells infected with the indicated viruses in (G). The results presented are from two biological replicates with at least 25 randomly chosen cells per treatment from each replicate. Outliers were excluded by IQR score. Molecular mass markers are indicated to the left of blots. One-way ANOVA was used to calculate statistical significance between samples. ns; not significant, *; *P* ≤ 0.05, **; *P* ≤ 0.01, and ***; *P* ≤ 0.001. The scale bar on images = 10 µm.

We next investigated whether TRIM25 ubiquitylation was evident in cells infected with VACV-Cop deletion viruses and other VACV strains. To address the former, HeLa cells were infected with VACV-Cop strains with large deletions in either the left (vP796), right (vP759), or both (vP811) arms of the virus genome (Fig. 6A) (37). Cells infected with either of the single-arm deletion viruses resulted in the formation of HMW, ubiquitylated, TRIM25 species, but this was not observed in cells infected with the vP811 virus (Fig. 6B and C). Furthermore, Western blotting analysis revealed ubiquitylated forms of TRIM25 were readily evident in HeLa cells infected with each of the VACV strains tested, except VACV-WR (Fig. 6D). In the latter, TRIM25 HMW bands were less evident, and very little decrease, if any, in the TRIM25 LMW band was observed (Fig. 6D).

We also examined whether infection of HeLa cells with other Orthopoxviruses promoted TRIM25 ubiquitylation. HMW TRIM25 species and a decrease in anti-TRIM25 immunoreactive bands were observed in cells infected with CPXV Brighton Red (CPXV-BR) strain (Fig. 6E and F) or MPXV-Clade IIb strain (Fig. S4). Interestingly, we observed a decrease in immunoreactive TRIM25 levels, but no HMW TRIM25 species, in HeLa cells infected with ectromelia virus-Moscow strain (ECTV-Mos) (Fig. 6E and F). Taken together, these results suggest that TRIM25 is ubiquitylated in response to infection of HeLa cells with many, but not all, Orthopoxviruses. Moreover, these findings are consistent with a gene(s) from either arm of the VACV-Cop genome being sufficient to induce TRIM25 ubiquitylation.

We next investigated whether the ability of other poxviruses to induce HMW, ubiquitylated TRIM25 also correlated with TRIM25 relocalization. Infection of HeLa cells with viruses able to induce TRIM25 HMW species (VACV-Cop, CPXV-BR) also induced the formation of TRIM25 punctae, whereas those that could not induce TRIM25 HMW species (vP811, ECTV-Mos) did not (Fig. 6G and H). Thus, even though the formation of TRIM25 punctae does not require ubiquitylation, there was a correlation between punctae formation and the appearance of TRIM25 ubiquitylated species.

### C16, a Bcl-2 family-like protein encoded by identical genes (C16L/B22R) on either end of the VACV-Cop genome, was necessary and sufficient to induce TRIM25 ubiquitylation and puncta formation

We reasoned that the gene responsible for promoting TRIM25 ubiquitylation and relocalization was likely one of the duplicated genes found on either end of the VACV-Cop genome. Therefore, we investigated whether there were any duplicated genes in VACV-Cop that were deleted in vP811, present in poxviruses that could induce TRIM25 ubiquitylation and relocalization (e.g., CPXV-BR), and absent in those that could not (e.g., ECTV-Mos). This led us to investigate the protein product of the C16L/B22R genes as a possible candidate. These genes at opposite ends of the genome encode for an identical Bcl-2 family-like protein that is expressed early during infection (40) and is also present in the virus particle (77). Moreover, C16/B22 (hereafter referred to as C16) was shown to act as a host range factor by counteracting the ZAP antiviral protein (78). Given that TRIM25 ubiquitylates and enhances ZAP activity (62), we examined whether C16 was responsible for TRIM25 ubiquitylation and relocalization.

**Fig 7:**
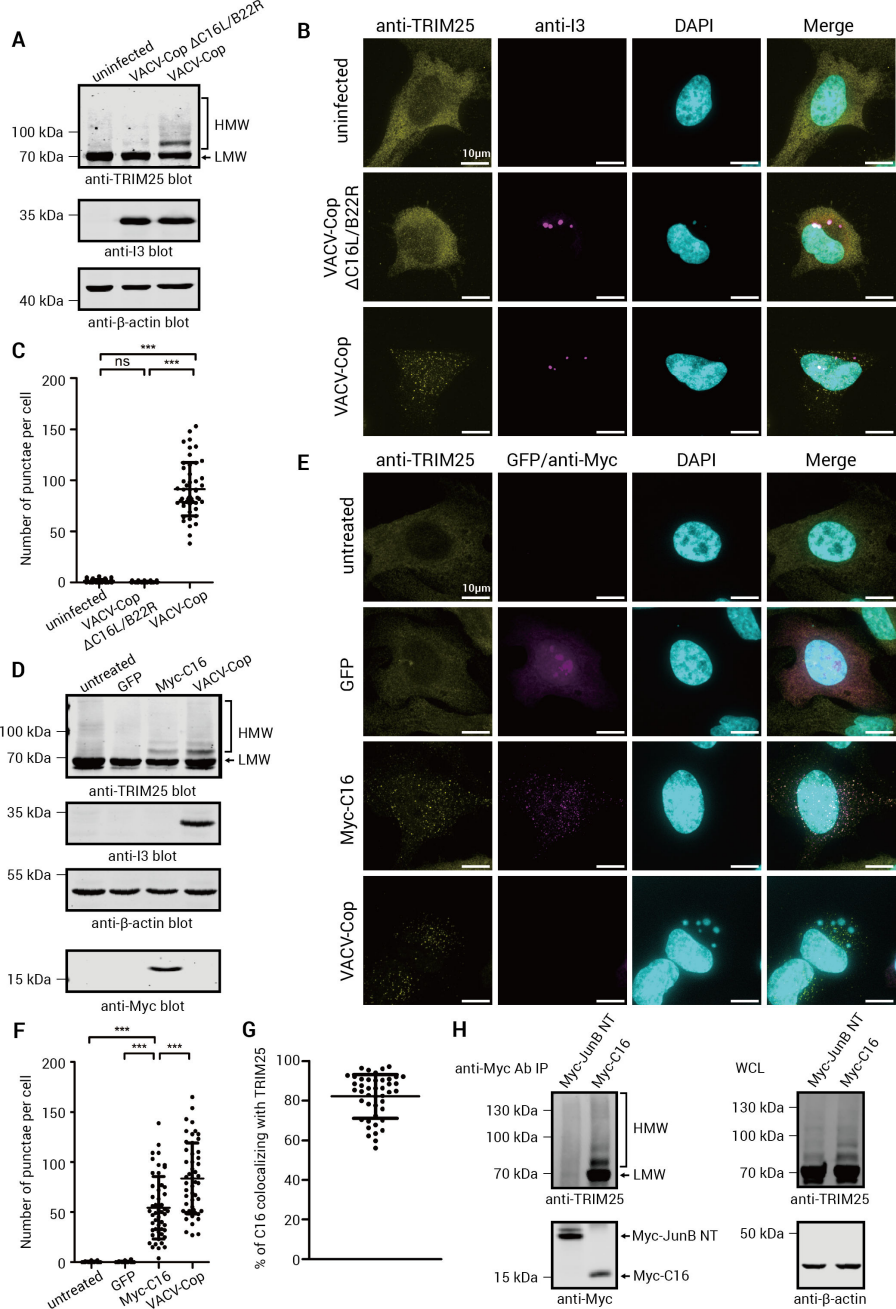
C16, the protein encoded by the VACV-Cop C16L/B22R genes, is necessary and sufficient to promote TRIM25 ubiquitylation and relocalization. (A) Lysates of HeLa cells either left uninfected or infected (MOI of 10) for 4 h with VACV-Cop or VACV-Cop ΔC16L/B22R were immunoblotted with Abs against the indicated proteins. (B) HeLa cells were either left uninfected or infected with VACV-Cop or VACV-Cop ΔC16L/B22R at an MOI of 3 for 4 h. Cells were then fixed, permeabilized, blocked, and stained with the antibodies against the indicated proteins. DAPI was used to stain DNA. (C) Quantification of the number of TRIM25 punctae/cell in VACV-Cop-uninfected, VACV-Cop-infected, or VACV-Cop ΔC16L/B22R-infected cells in (B). The results presented are from two biological replicates with at least 25 randomly chosen cells per treatment from each replicate. Outliers were excluded by IQR score. (D) Lysates of uninfected, VACV-infected (4 h), or HeLa cells transfected overnight with plasmids encoding for GFP (control) or Myc-C16 were immunoblotted with Abs against the indicated proteins. Note: The anti-Myc blot was from a different membrane probing the same samples. (E) HeLa cells were either left untreated, infected for 4 h with VACV-Cop, or transfected overnight with plasmids encoding for GFP or Myc-C16. Cells were then fixed, permeabilized, blocked, and stained with the antibodies against the indicated proteins. DAPI was used to stain DNA. (F) Quantification of the number of TRIM25 punctae/cell in GFP- or Myc-C16-transfected HeLa cells in (E). The results presented are from two biological replicates with at least 25 randomly chosen cells per treatment from each replicate. Outliers were excluded by IQR score. (G) Examination of Myc-C16 and TRIM25 colocalization in HeLa cells transfected with a plasmid coding for Myc-C16. (H) Cell lysates from HeLa cells transfected with the plasmid encoding the Myc-JunB N-terminal fragment (Myc-JunB NT) or Myc-C16 were used to perform IPs with an anti-Myc Ab. IPs were then Western blotted with an anti-TRIM25 Ab (upper panel) and with the anti-Myc Ab (lower panel). Whole-cell lysate blots are shown on the left. The scale bar on images = 10 µm.

We generated a VACV-Cop strain lacking C16L and B22R genes (VACV-Cop ΔC16L/B22R) and found that HeLa cells infected with this virus were unable to induce the formation of TRIM25 HMW, ubiquitylated species (Fig. 7A) nor relocalize TRIM25 to punctate structures (Fig. 7B and C). In addition, we found that the expression of an Myc-tagged C16 protein in HeLa cells was sufficient to promote the formation of TRIM25 HMW species (Fig. 7D) and TRIM25 punctae (Fig. 7E and F). Moreover, Myc-tagged C16 colocalized with TRIM25 punctae (Fig. 7E and G). Since we observed the colocalization of C16 and TRIM25 by immunofluorescence, we investigated whether the proteins co-IPed. TRIM25, including HMW species, was observed in anti-Myc C16 IPs, but not IPs with an irrelevant Myc-tagged protein (Fig. 7H). Collectively, these experiments demonstrate that C16 is both necessary and sufficient to promote TRIM25 ubiquitylation and relocalization and that C16 and TRIM25 interact with each other.

### Loss of TRIM25 did not significantly alter the growth rate of VACV-Cop, vP811, or VACV-Cop ΔC16L/B22R

**Fig 8:**
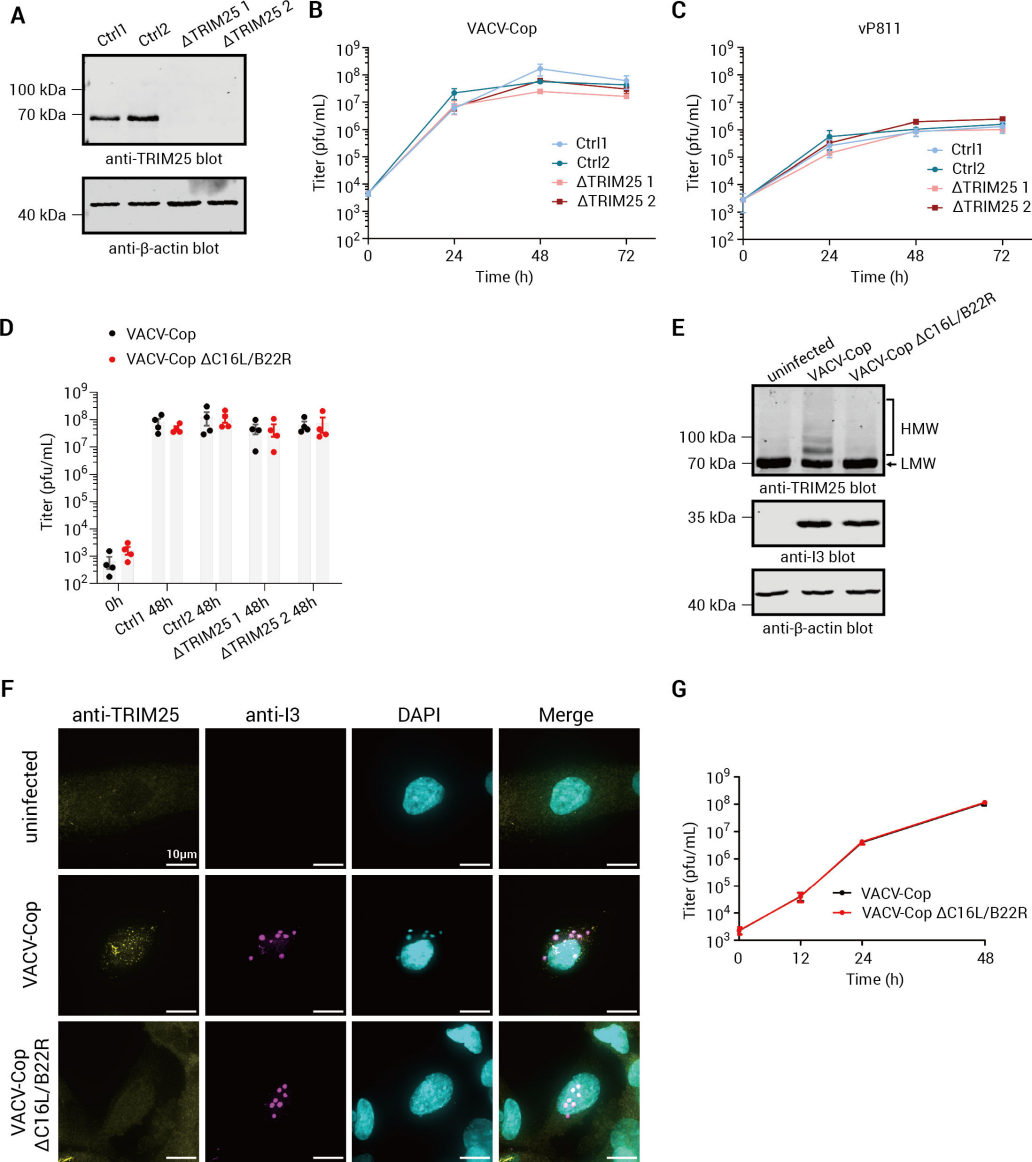
The absence of TRIM25 in HeLa cells does not alter the replication of VACV-Cop, vP811, or VACV-Cop ΔC16L/B22R. (A) Immunoblot analysis showing the absence of TRIM25 in two knock-out HeLa cell clones (generated with the same crRNA) and the presence in clones from cells electroporated with a non-targeting (control) guide RNA. Molecular mass markers are indicated to the left of blots. Viral titers were determined from HeLa cells with or without TRIM25, which were infected with VACV-Cop (B) or vP811 (C) at an MOI of 0.03 for the indicated time points. The data represent the average and standard error of the mean of four independent experiments. A two-way ANOVA found that there was no statistically significant difference between the titer of virus in ΔTRIM25 and control cells at each of the time points post-infection. (D) Viral titers were determined from HeLa cells with or without TRIM25, which were infected with VACV-Cop or VACV-Cop ΔC16L/B22R at an MOI of 0.03 for 48 h. The data represent the average and standard error of the mean of four independent experiments. A two-way ANOVA found that there was no statistically significant difference between the titer of viruses in ΔTRIM25 and control cells. Western blot analysis (E) and puncta formation (F) showing TRIM25 HMW species and puncta formation in VACV-Cop-, but not VACV-Cop ΔC16L/B22R-infected, HFFF-TERT cells. (G) Viral titers were determined from HFFF-TERT cells, which were infected with VACV-Cop or VACV-Cop ΔC16L/B22R at an MOI of 0.03 for the indicated time points. The data represent the average and standard error of the mean of four independent experiments. A two-way ANOVA found that there was no statistically significant difference between the titers of the two viruses. The scale bar on images = 10 µm.

Many members of the TRIM superfamily are associated with innate immune signaling and restricting virus infection (79). For example, TRIM5α was demonstrated to restrict Orthopoxvirus infection, and VACV overcomes this restriction factor, in part, by promoting TRIM5α ubiquitylation and degradation (80). Therefore, Ub-mediated degradation of TRIM family proteins is one strategy used by poxviruses to evade the host immune response in order to efficiently replicate in cells. We postulated that the formation of HMW, ubiquitylated species, and/or the relocalization of TRIM25 could be mechanisms for VACV-Cop to overcome the antiviral activity of TRIM25. Because vP811 and VACV-Cop ΔC16L/B22R cannot induce TRIM25 ubiquitylation or relocalization, we hypothesized that TRIM25 may restrict the replication of these viruses. To test this hypothesis, we generated TRIM25 knock-out HeLa cell lines using CRISPR-/Cas9-mediated gene editing (Fig. 8A). We then examined the growth of VACV-Cop, vP811, and VACV-Cop ΔC16L/B22R in two control and TRIM25 knock-out cell lines. Overall, there was no difference in the growth rate of either VACV-Cop (Fig. 8B) or vP811 (Fig. 8C) in control or TRIM25 knock-out HeLa cells. Moreover, VACV-Cop and VACV-Cop ΔC16L/B22R titers at 48 hpi were comparable between TRIM25 knock-out and control cell lines (Fig. 8D). Since antiviral pathways can be altered in transformed cells (81), we examined whether replication of VACV-Cop ΔC16L/B22R was impaired in a non-transformed cell line. Infection of HFFF-TERT cells with VACV-Cop resulted in the formation of HMW, ubiquitylated TRIM25 species (Fig. 8E), and TRIM25 punctae (Fig. 8F), but these were not observed in cells infected with VACV-Cop ΔC16L/B22R. However, no significant difference in replication of these two viruses was observed in HFFF-TERT cells (Fig. 8G). Thus, TRIM25 deletion in HeLa cells is not sufficient to confer a growth advantage for viruses that cannot ubiquitylate or relocalize TRIM25 nor is the deletion of C16L/B22R sufficient to impair the replication of VACV-Cop in HFFF-TERT cells.

## DISCUSSION

In this study, we examined how the UPS is engaged early after VACV-Cop infection to reveal novel ways that poxviruses exploit this system for their benefit. To that end, we demonstrated that the antiviral E3 Ub/ISG15 ligase, TRIM25, was ubiquitylated and relocalized to punctate structures in VACV-Cop-infected cells in a manner dependent on the viral protein, C16. We propose that this may represent a strategy for poxviruses to evade the antiviral activities of TRIM25.

To examine changes in ubiquitylation during infection, we enriched for peptides with a diGly Ub remnant motif in order to identify both degradation-dependent and -independent ubiquitylation events (47, 48). The most remarkable observation from these experiments was the number of TRIM25 diGly peptides either enriched for or exclusively found in VACV-Cop-infected HeLa cells (Fig. 1C). Of note, a study using diGly enrichment to identify ubiquitylated proteins in mature CPXV-BR virions with a limited examination of cellular protein ubiquitylation during CPXV-BR infection of HeLa cells identified diGly peptides from TRIM25, as well as several other proteins identified in our study (23). The finding that infection with these two Orthopoxviruses induced a similar set of diGly peptides supports the notion that different poxviruses engage the UPS in similar ways.

To further examine the impact of VACV-Cop infection on TRIM25, we performed Western blotting experiments, which showed that TRIM25 forms HMW, ubiquitylated species in VACV-Cop-infected cells (Fig. 2B and Fig. 3A). Proteasomal or lysosomal degradation may not be a significant consequence of TRIM25 ubiquitylation (**Fig. 4**), and we hypothesize that the decrease in anti-TRIM25 immunoreactive bands in infected cells (Fig. 2B and C) is, at least partially, due to the anti-TRIM25 Ab having reduced affinity for the modified protein. Therefore, we investigated whether TRIM25 ubiquitylation may serve another purpose and found that this correlated with relocalization of the protein to punctate structures in infected cells (Fig. 5). These two observations are strikingly similar to the strategy used by Epstein-Barr virus (EBV) to evade the antiviral activities of TRIM25. The EBV protein, BPLF1, complexes with TRIM25 and cellular 14-3-3 proteins (82). This promotes TRIM25 autoubiquitylation and sequesters the protein away from RIG-I in protein aggregates that stain positive for the autophagy marker, p62/SQSTM1 (61). While the TRIM25-positive punctae in VACV-Cop-infected HeLa cells do not appear to be p62-/SQSTM1-positive (results not shown), we hypothesize that VACV-Cop utilizes a similar strategy to overcome the antiviral activities of TRIM25. The identification of these punctate structures will be important to test this hypothesis. In addition, we initially posited that relocalization of TRIM25 was a consequence of its ubiquitylation, but this does not appear to be the case as TRIM25 relocalization still occurred when cellular ubiquitylation was inhibited (Fig. 5). We suspect that TRIM25 ubiquitylation may be a consequence of the relocalization and that TRIM25 may be autoubiquitylating itself.

We also identified the viral protein responsible for the ubiquitylation and relocalization of TRIM25. Using large deletion strains of VACV-Cop, we determined that a gene(s) at either end of the genome was sufficient to induce TRIM25 ubiquitylation and relocalization (Fig. 6). Poxviruses have many duplicated genes at the ends of their genomes (83), and we investigated the C16 protein, which is encoded by the C16L and B22R genes, as a possible candidate. We found that C16 was both necessary and sufficient to induce TRIM25 ubiquitylation and relocalization and that C16 associates with TRIM25 (Fig. **7**). It is important to note that C16 is a Bcl-2 family-like protein that lacks obvious E3 Ub-ligase activity. Therefore, C16 likely promotes TRIM25 ubiquitylation either through enhancing TRIM25 autoubiquitylation activity or recruiting a cellular E3 Ub-ligase to ubiquitylate TRIM25.

C16 was previously identified as a VACV host range factor (40, 78) through interfering with the ZAP antiviral protein (78). As well, TRIM25 binds and activates ZAP through forming K63- and K48-linked polyubiquitin chains on ZAP (62), and activated ZAP contributes to the antiviral response by degrading viral RNAs and inhibiting the translation of viral proteins (62, 84, 85). Peng et al. demonstrated that adding back C16L into modified VACV Ankara (MVA), a large VACV deletion virus that lacks the genes encoding for C16, improved MVA replication in human cell lines via an undefined mechanism that enhanced virion morphogenesis (40, 78). Moreover, similar to the relocalization of TRIM25 by C16 (Fig. 5), infection of A549 cells with MVA reconstituted with C16 resulted in the formation of ZAP punctae that colocalized with C16 (78), although it did not appear to affect the colocalization of TRIM25 and ZAP (78). Thus, how significantly TRIM25 and ZAP punctae overlap and whether C16 interacts with one of the proteins to recruit the other to punctate structures are unclear and require further investigation.

Because ZAP is a host restriction factor that is overcome by C16 (78), we investigated whether TRIM25 was also a host restriction factor antagonized by C16. We found no growth advantage for VACV-Cop ΔC16L/B22R or vP811 in TRIM25 knock-out (ΔTRIM25) HeLa cells (Fig. 8). In addition, to rule out these observations were due to an impairment in the antiviral response in HeLa cells, we performed growth curve experiments with non-transformed HFFF-TERT cells and also found no impairment in the replication of VACV-Cop ΔC16L/B22R in these cells (Fig. 8G). Collectively, we believe these findings are not surprising given that poxviruses have multiple strategies to suppress antiviral signaling pathways downstream of TRIM25. For example, VACV C6 inhibits IFN expression by blocking IRF3 activation and nuclear translocation during poxvirus infection (86). IRF3 activation is also inhibited by the VACV N1 protein interacting with the IKK complex and suppressing NF-κB signaling (87). NF-κB signaling is also inhibited by VACV A49 mimicking cellular IκBα and preventing IκBα degradation, thereby blocking the nuclear translocation of p65 NF-κB (88). Thus, additional mechanisms possessed by poxviruses to inhibit signaling events downstream of TRIM25 are very likely compensating for the loss of TRIM25. To overcome these potential complications, experiments taking a more reductionist approach using MVA expressing C16, or the C16 protein, alone need to be performed to definitively determine whether TRIM25 is a restriction factor overcome by C16. In addition, while deletion of C16L/B22R did not inhibit VACV-Cop replication in the cell lines tested in this study, these findings do not preclude C16 from being an important virulence factor *in vivo*.

While not all Orthopoxviruses possess C16, our data suggests that these viruses may have alternative ways of antagonizing TRIM25. In ECTV-infected HeLa cells, we did not observe HMW, ubiquitylated species nor relocalization of TRIM25 into punctae, but we observed a decrease in TRIM25 levels (Fig. 6E through H). Reduced TRIM25 levels were also observed in HFFF-TERT cells infected with VACV-WR, which also lacks C16, and these levels were partially rescued by treatment with MG132 (Fig. 1E) (39).

In addition to the TRIM25 diGly peptides, we identified many other diGly peptides from intriguing cellular proteins including those previously implicated in poxvirus infection. For example, we identified an IFIT1 and/or IFIT5 diGly peptide in VACV-Cop-infected cells. The IFITs are a family of antiviral factors that are induced by interferon and can inhibit the transcription of viral RNAs (89). IFITs are recognized by the VACV C9 protein, which is an ANKR/F-box-containing substrate adapter that co-opts cellular multi-subunit E3 Ub-ligase components, to promote the Ub-mediated degradation of IFITs and enhance viral DNA replication (28). As well, we identified five diGly peptides from the RACK1 scaffolding protein, of which one (K175) was exclusively found in VACV-Cop-infected cells (Fig. 1C). RACK1 performs many functions, one of which is to regulate translation as a component of ribosomes (50, 90). RACK1 is phosphorylated by the VACV-encoded B1 kinase to selectively increase translation of viral RNAs (91). Of note, RACK1 protein levels, and several other proteins for which we identified diGly peptides, did not decrease in VAV-WR-infected HFFF-TERT cells (Fig. 1E) (39). This suggests that ubiquitylation of these proteins could perform a regulatory rather than a degradatory function; however, we cannot rule out that the identified diGly peptides represent a small pool of the respective proteins that are targeted for degradation.

While we were primarily interested in the diGly peptides derived from cellular proteins, we identified many from viral proteins. These included peptides from proteins associated with virus genome replication, transcription/translation, immune evasion, and blocking programmed cell death (Fig. 1B). Whether ubiquitylation of these proteins is a means to regulate their activity or simply ubiquitylated defective ribosomal products from misfolded viral proteins (92) or damaged viral proteins remains to be determined.

In conclusion, many viral proteins interfere with the antiviral activity of TRIM25. The EBV protein, BPLF1, relocalizes the protein away from RIG-I (61), and influenza A virus NS1 protein binds to the TRIM25 coiled-coil domain, which blocks TRIM25 from multimerizing and ubiquitylating RIG-I (56). Similarly, the SARS-CoV nucleocapsid protein binds the TRIM25 PRY/SPRY domain and inhibits TRIM25 from interacting with and ubiquitylating RIG-I (56). The results of this study identify two putative C16-dependent and C16-independent mechanisms by which poxviruses could interfere with TRIM25 antiviral activity.

## Data Availability

Mass spectrometry data files are available through the MassIVE Repository, MSV000094020. Reasonable requests for other data and materials can be made to the authors.

## SUPPLEMENTAL MATERIAL

Figure S1 jvi.00898-25-s0001.tif Comparison of diGly peptides found in individual experiments.

Figure S2 jvi.00898-25-s0002.tif Analysis of pathways and processes associated with proteins with diGly peptides enriched in infected or uninfected HeLa cells.

Figure S3 jvi.00898-25-s0003.tif Anti-TRIM25 IPs and anti-Ub blots of uninfected or VACV-Cop-infected HeLa cell lysates.

Figure S4 jvi.00898-25-s0004.tif TRIM25 HMW, ubiquitylated species were observed in HeLa cells infected with MPXV.

Supplemental legends jvi.00898-25-s0005.docx Legends for all supplemental material.

Supplemental tables jvi.00898-25-s0006.xlsx Tables SI to SIII.

ASM does not own the copyrights to Supplemental Material that may be linked to, or accessed through, an article. The authors have granted ASM a non-exclusive, world-wide license to publish the Supplemental Material files. Please contact the corresponding author directly for reuse.
