# Supplementary figures and images for: The vaccinia virus protein, C16, promotes the ubiquitylation and relocalization of the antiviral E3 ubiquitin-ligase, TRIM25
Source: J Virol. 2025 Jul 28;99(8):e00898-25. doi: 10.1128/jvi.00898-25 (PMC12363216; doi:10.1128/jvi.00898-25)

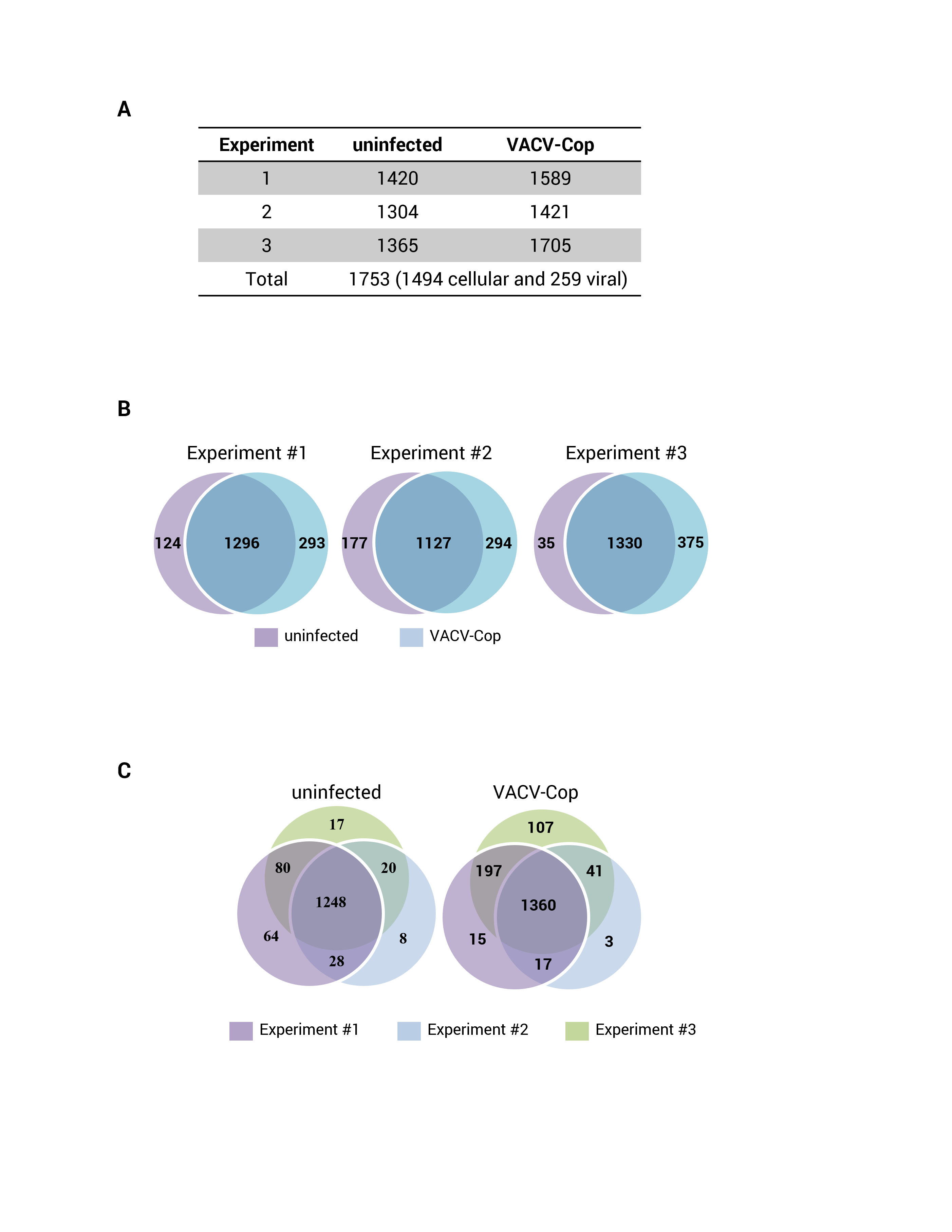

Supplement: Figure S1 — Comparison of diGly peptides found in individual experiments. [file jvi.00898-25-s0001.tif]

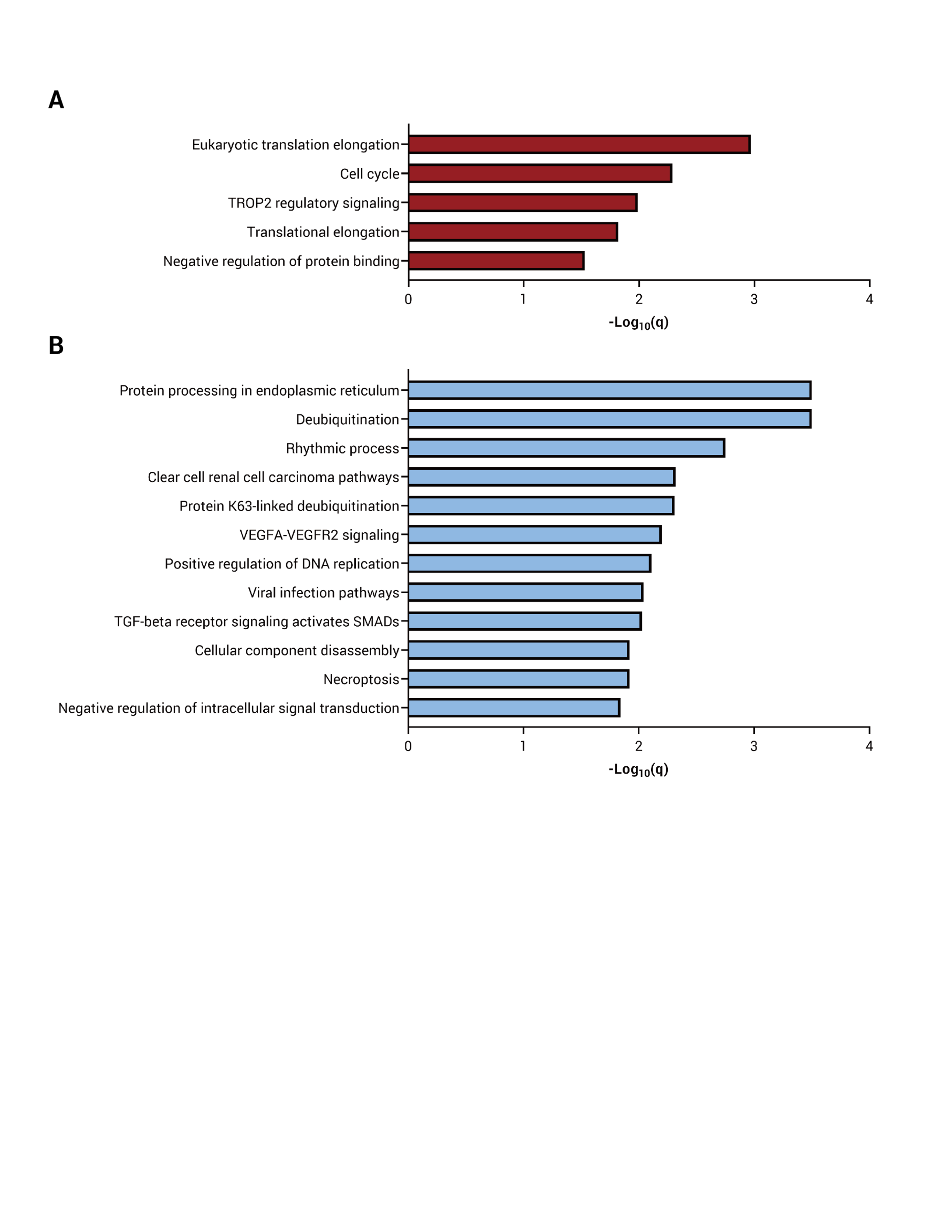

Supplement: Figure S2 — Analysis of pathways and processes associated with proteins with diGly peptides enriched in infected or uninfected HeLa cells. [file jvi.00898-25-s0002.tif]

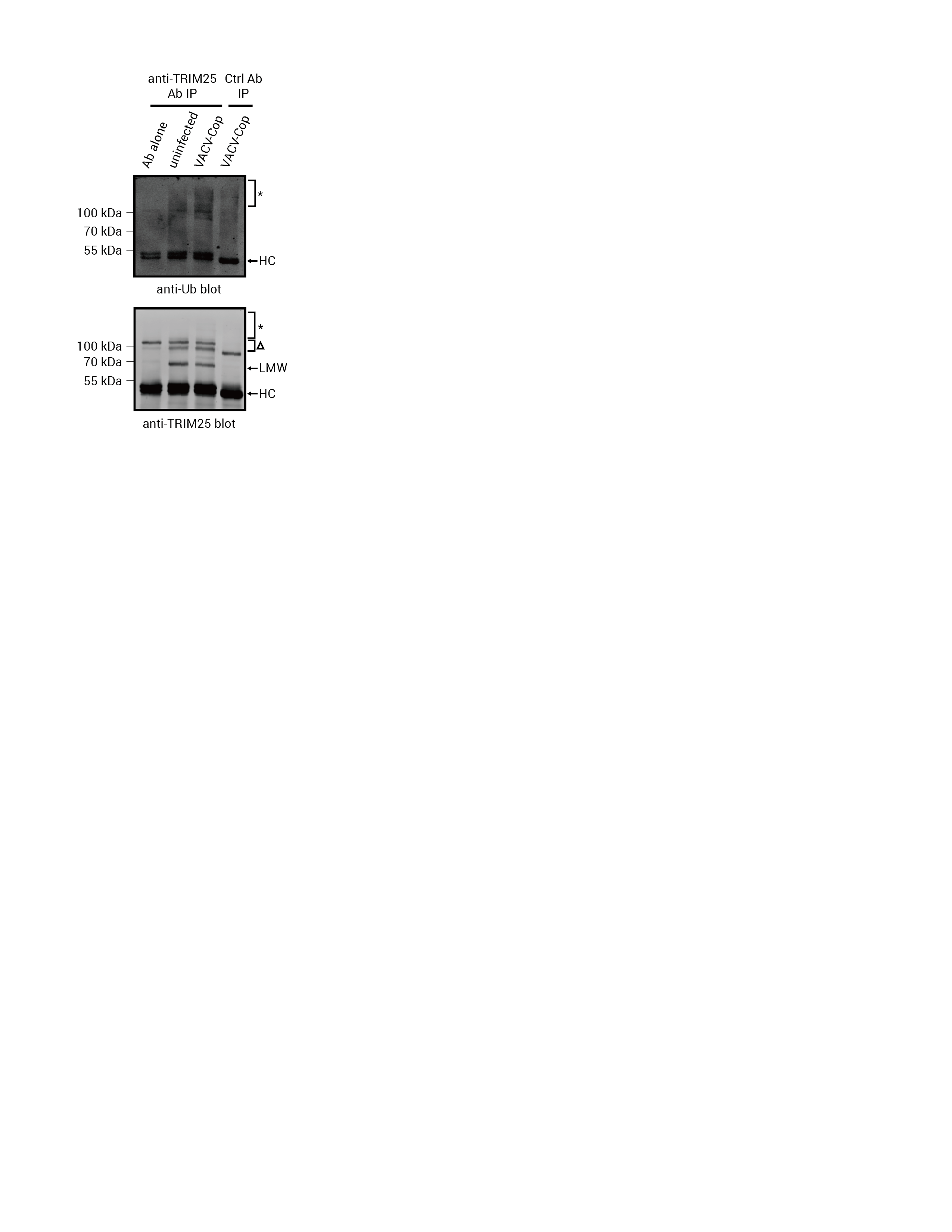

Supplement: Figure S3 — Anti-TRIM25 IPs and anti-Ub blots of uninfected or VACV-Cop-infected HeLa cell lysates. [file jvi.00898-25-s0003.tif]

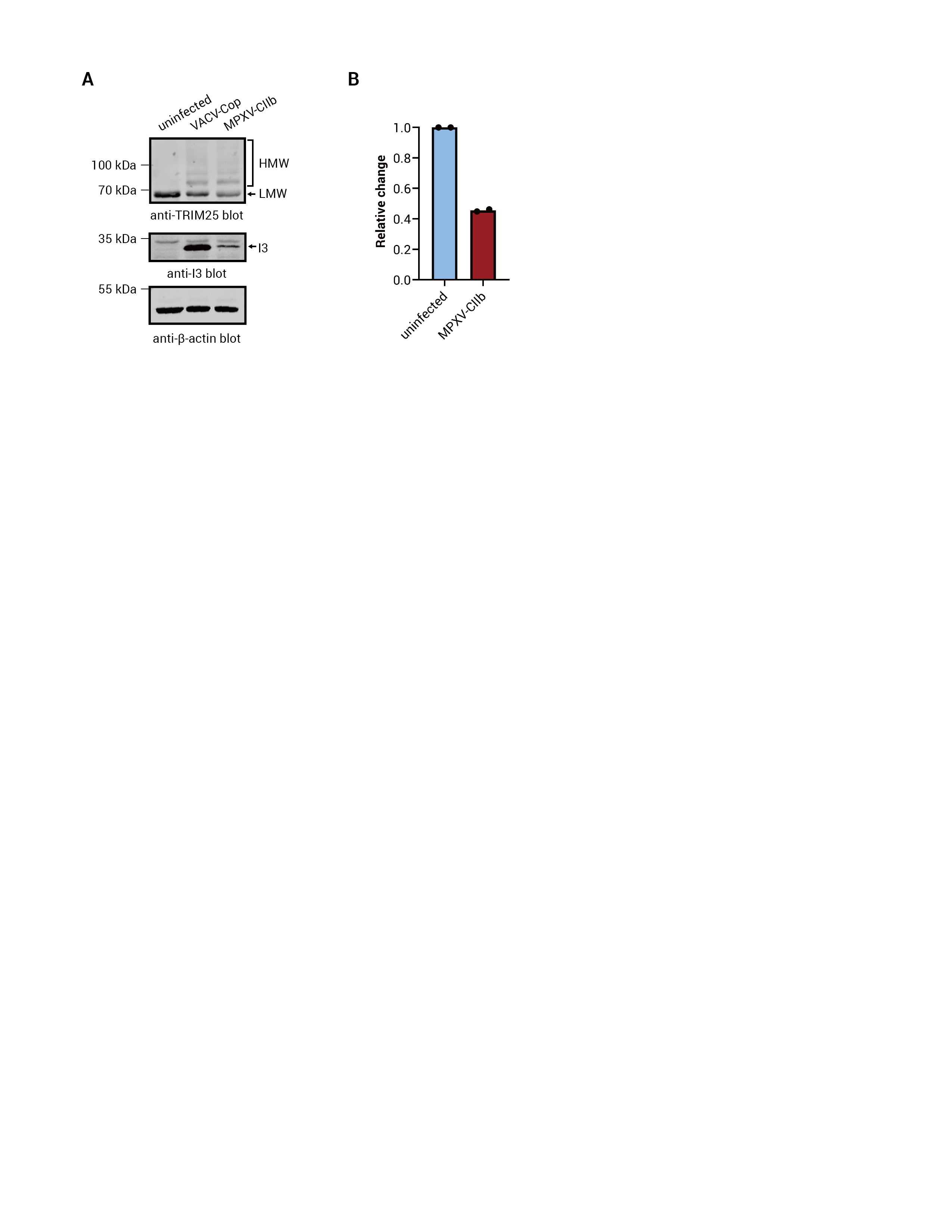

Supplement: Figure S4 — TRIM25 HMW, ubiquitylated species were observed in HeLa cells infected with MPXV. [file jvi.00898-25-s0004.tif]
