# Supplementary material for: The vaccinia virus protein, C16, promotes the ubiquitylation and relocalization of the antiviral E3 ubiquitin-ligase, TRIM25
Source: J Virol. 2025 Jul 28;99(8):e00898-25. doi: 10.1128/jvi.00898-25 (PMC12363216; doi:10.1128/jvi.00898-25)
Supplement: Supplemental legends — Legends for all supplemental material. [file jvi.00898-25-s0005.docx]

**Supplementary Figure and Table legends**

**Supplementary Figure 1 - Comparison of diGly peptides found in individual experiments. A,** Table listing the number of the peptides identified in uninfected cells and VACV-Cop-infected cells in each independent experiment. **B,** Venn diagrams illustrating the number of the peptides identified in uninfected or infected cells in common in each of the three independent experiments. **C,** Summary of overlapping diGly peptides identified in the three independent experiments for infected and uninfected cells.

**Supplementary Figure 2 – Analysis of pathways and processes associated with proteins with diGly peptides enriched in infected or uninfected HeLa cells.** Metascape analysis of proteins associated with diGly peptides enriched VACV-Cop-infected **(A)** or uninfected **(B)** HeLa cells in at least 2 independent experiments. Categories with significant adjusted *p* value ((-log(q) > 1.5 or q < 0.05) are shown.

**Supplementary Figure 3 – Anti-TRIM25 IPs and anti-Ub blots of uninfected or VACV-Cop-infected HeLa cell lysates.** Cell lysates from uninfected HeLa cells or HeLa cells infected for 4h with VACV-Cop (MOI of 10) were used to perform IPs with either an anti-TRIM25 Ab or isotype control (anti-HJURP) Ab. Anti-TRIM25 IPs with the lysate omitted (Ab alone) were performed to identify bands derived from the anti-TRIM25 Ab. IPs were then western blotted with an anti-Ub Ab (upper panel) before being reprobed with the anti-TRIM25 Ab (lower panel). Non-specific bands are indicated by Δ and putative ubiquitylated forms of TRIM25 were indicated by an *; HC; heavy chain of the Ab used for IP.

**Supplementary Figure 4 – TRIM25 HMW, ubiquitylated species were observed in HeLa cells infected with MPXV.**  **A,** Lysates of HeLa cells either left uninfected or infected (MOI of 10) for 4h with the indicated viruses were immunoblotted with Abs against the indicated proteins. **B,** Quantification of total anti-TRIM25 immunoreactive bands in **A**. Quantification is from 2 independent experiments and expressed relative to the samples left not infected with viruses.

**Supplementary Table I – Identified diGly peptides.** List of cellular and viral diGly peptides identified in at least one of the independent experiments.

**Supplementary Table II – Enriched diGly peptides in VACV-Cop-infected cells.** List of cellular and viral diGly peptides identified as up-regulated (≥ 2-fold change and *p* ≤ 0.05) in at least two independent experiments.

**Supplementary Table III – Enriched diGly peptides in uninfected cells.** List of cellular diGly peptides identified as down-regulated (≥ 2-fold change and *p* ≤ 0.05) in at least two independent experiments.
